# Investigating the causal impact of gut microbiota on periodontitis based on Mendelian randomization, bulk transcriptomics, and single-cell transcriptomics

**DOI:** 10.3389/fcell.2026.1884760

**Published:** 2026-08-19

**Authors:** Huiru Li, Runze Kong, Ping Chang, Fangyi Xie, Tianxiao Zhu, Yang Xie

**Affiliations:** 1 Department of Stomatology, The First Medical Center, Chinese PLA General Hospital, Beijing, China; 2 Department of Otolaryngology Head and Neck Surgery, Tangdu Hospital, Fourth Military Medical University, Xi’an, Shaanxi, China

**Keywords:** gut microbiota, mendelian randomization, oral-gut axis, periodontitis, single-cell transcriptome

## Abstract

**Background:**

Dysbiosis of the gut microbiota is closely associated with periodontitis (PD), yet whether a direct causal relationship exists and the underlying molecular mechanisms remain unclear. This study aims to identify key genes linking gut microbiota to PD, providing molecular targets for its precise prevention, diagnosis, and treatment.

**Methods:**

Three PD-related transcriptomic datasets (GSE10334, GSE223924, GSE171213) were obtained from public databases. Through Mendelian randomization (MR), machine learning, and expression validation, key regulatory genes causally linking gut microbiota to PD were identified. Gingival tissue specimens from PD patients (n = 6) and healthy controls (n = 6) were used for independent validation via quantitative real-time PCR(qRT-PCR) and Western blot. Immune cell infiltration analysis and cell communication network analysis were further conducted using single-cell RNA sequencing data.

**Results:**

MR analysis identified 22 gut microbiota taxa causally associated with PD (12 protective, OR<1; 10 risk-associated, OR>1). Transcriptomic analysis yielded seven candidate genes, which were narrowed to 4 feature genes via multi-algorithm machine learning. Cross-dataset validation confirmed three key genes—IL-19, NID2, and SH3D19—with consistent differential expression. Independent experimental validation confirmed significant upregulation of NID2 at both mRNA and protein levels in the PD group (*P* < 0.01), while IL-19 and SH3D19 showed no statistically significant differences. Immune infiltration analysis revealed increased plasma cell infiltration and decreased CD8^+^ T cell infiltration in PD (*P* < 0.05). Single-cell communication analysis showed that in controls, monocyte–neutrophil interactions predominated, whereas in PD, interactions along the monocyte/MDSC/neutrophil axis were additionally elevated.

**Conclusion:**

This multi-omics study identified 22 gut microbiota taxa causally associated with PD, and IL-19, NID2, and SH3D19 as key dysregulated genes. Among these, NID2 emerged as a prioritized candidate linking gut dysbiosis to periodontitis, warranting further functional investigation. These findings provide molecular evidence for the oral-gut axis and offer potential biomarkers and therapeutic targets for PD.

## Graphical Abstract

**Figure:**
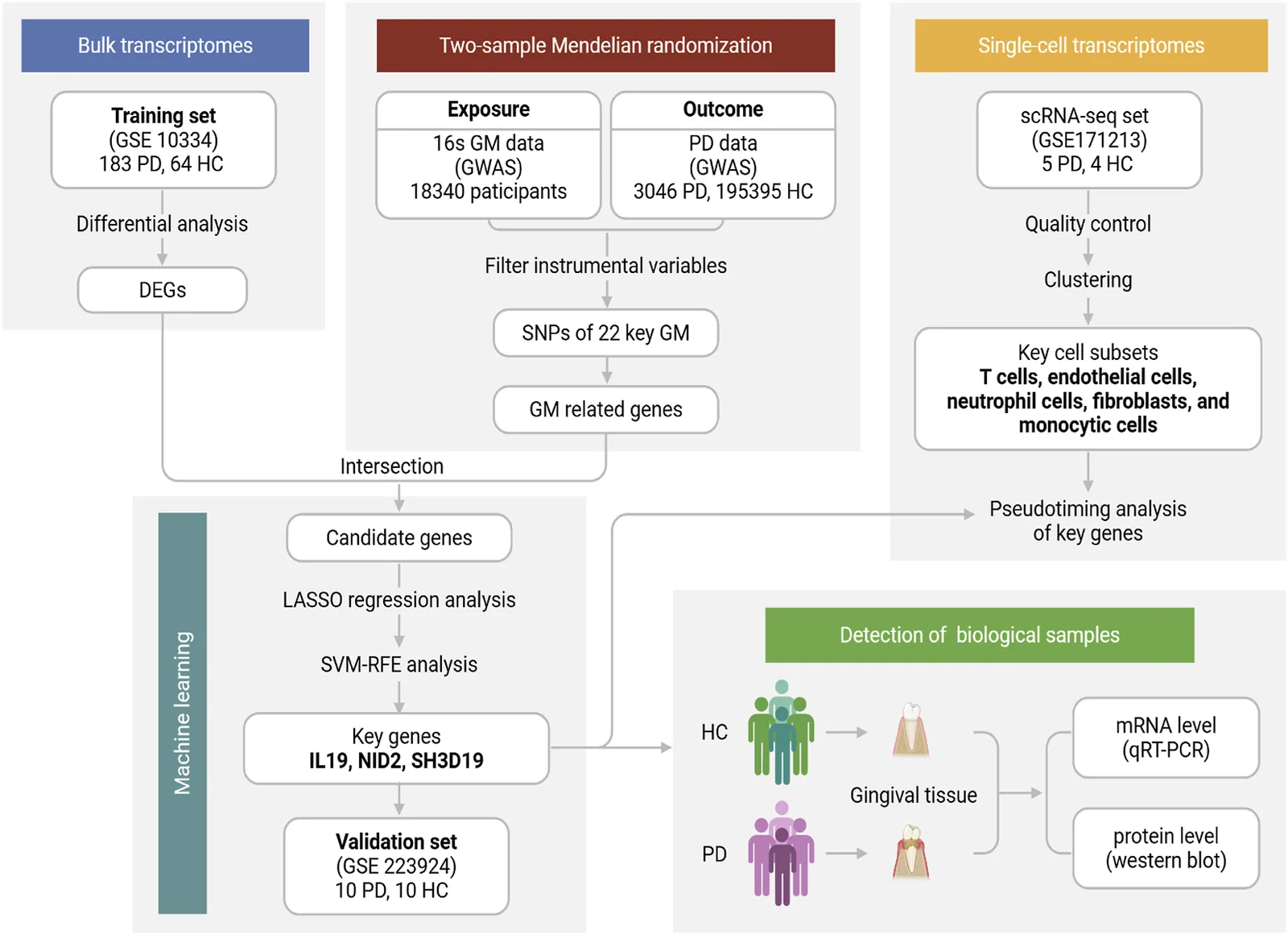

## 1 Introduction

Periodontitis (PD) represents a prevalent long-term inflammatory condition affecting the oral cavity, with a global prevalence of approximately 60%–70% in adults and an incidence that increases significantly with age (Tian et al., 2022). PD primarily affects the tooth-supporting structures, including the gums, periodontal ligament, alveolar bone, and cementum (Duspara et al., 2024). Clinical manifestations of PD include gingival recession, tooth loosening, and even tooth loss (Dwiputri et al., 2023). PD not only impairs oral health but is also associated with systemic diseases such as cardiovascular disease, diabetes, and rheumatoid arthritis through the release of inflammatory cytokines (Penmetsa et al., 2023). Traditionally, it is reported to begin with the overproliferation of pathogenic microorganisms in dental plaque biofilms, whose secreted virulence factors trigger abnormal host immune responses, ultimately leading to irreversible tissue damage (Dolinska et al., 2022). Recent advancements in holistic medicine have revealed PD as a network disease involving multifactorial interactions, including local factors, systemic factors, and microbial infections (Curtis et al., 2020). Investigating systemic regulatory mechanisms that affect PD is critical for achieving personalized treatment and reducing the risks associated with systemic diseases.

The gut microbiota (GM), commonly known as the “human second genome,” serves a vital function in influencing host health through metabolic regulation, immune training, and barrier maintenance (Yin et al., 2022). Recent studies highlight the oral-gut axis as a bidirectional bridge between PD and GM. On the one hand, PD-associated pathogens (e.g., Porphyromonas gingivalis) can migrate to the gut via the bloodstream, causing gut dysbiosis characterized by reduced beneficial bacteria (e.g., Bifidobacterium) and enrichment of pathogenic bacteria (e.g., Enterobacteriaceae) (Alyousef et al., 2023; Li et al., 2022a). On the other hand, GM metabolites (e.g., short-chain fatty acids, SCFAs) may regulate the local immune microenvironment in periodontal tissues via the circulatory system (Zhao et al., 2021). However, existing research is mainly limited to correlation analyses between GM and PD. Recent Mendelian randomization (MR) studies have begun exploring the causal relationship between GM and PD. However, most analyses are restricted to taxonomic profiling and fail to identify downstream molecular mediators (Ye et al., 2023; Yu et al., 2025). The key functional strains and their underlying molecular mechanisms remain unclear.

MR is a causal inference method based on genetic variation that can effectively mitigate confounding biases in traditional observational studies, which can provide high-level evidence for the causal relationship between GM and PD (Wang et al., 2024). Methodologically, our MR analysis incorporates a systematic SNP-to-gene mapping step to translate genetic instruments into candidate gene sets—a strategy not previously applied in GM-PD MR studies. To our knowledge, this is the first study that integrates MR, bulk transcriptomics, machine learning, single-cell RNA sequencing (scRNA-seq), and experimental validation to systematically investigate the causal mechanisms by which GM affects PD—encompassing population-level causal associations, single-cell precision molecular mechanisms and independent experimental verification at both the transcriptional and protein levels. Firstly, we employed a two-sample MR analysis to screen for GM taxa causally linked to PD, based on publicly available databases. Furthermore, core genes associated with periodontitis were identified through differential expression gene analysis. To validate the differential expression of key genes screened via bioinformatics at both transcriptional and protein levels, quantitative detection of candidate gene expression in gingival tissues from periodontitis patients and healthy controls was performed using qRT-PCR and Western blot, thereby supporting the reliability and clinical relevance of the analytical conclusions. Further elucidation of their pathological roles was achieved through functional enrichment and immune infiltration analyses. Additionally, single-cell RNA sequencing data were utilized to determine the distribution patterns of key genes across cellular subpopulations within periodontal tissues. This multi-omics framework deepens the understanding of the oral-gut axis and provides novel strategies for the precise intervention of periodontitis.

## 2 Materials and methods

### 2.1 Data collection

The GM data were from the GWAS dataset of MiBioGen (www.mibiogen.org). This dataset included whole-genome genotype data and 16S fecal microbiota data from a total of 18,340 participants across 24 cohorts in 11 countries (Kurilshikov et al., 2021). Moreover, genome-wide association study (GWAS) data for PD (finn-b-K11_PERIODON_CHRON) were retrieved using the IEU Open GWAS database, which included 198,441 European samples (3,046 cases and 195,395 control samples) and 16,380,378 single-nucleotide polymorphisms (SNPs).

Furthermore, PD-related datasets were obtained from the Gene Expression Omnibus (GEO) repository. Among them, the bulk transcriptome dataset GSE10334 (platform GPL570) was composed of gingival tissue specimens from 183 PD patients and 64 controls, which were designated as a training set. The validation set GSE223924 (platform GPL24676) encompassed gingival tissue samples from 10 PD patients and 10 controls (10 peri-implantitis samples were excluded). In addition, the single-cell dataset GSE171213 (GPL24676) incorporated gingival tissue samples from 5 PD patients and 4 controls (3 samples after treatment were excluded).

### 2.2 Filter of the instrumental variables (IVs)

To investigate the GM with causal relationships to PD, MR methodology was applied, employing GM as the exposure measure and PD as the outcome measure. Additionally, to obtain dependable results, MR analysis fulfilled three core assumptions: Firstly, genetic variation in IVs should be highly correlated with exposure factors; secondly, genetic variation in IVs should not be associated with confounding factors; and thirdly, genetic variation in IVs affects outcome variables only through exposure factors. To select IVs demonstrating significant correlation with exposure variables and to minimize linkage disequilibrium-related bias, the extract_instruments function within the ‘TwoSampleMR’ package (v 0.6.6) [13] was applied to process exposure data and identify appropriate IVs (P < 1 × 10^−5^, clump = TRUE, *R* ^2^ = 0.001, window size = 100 kb), a threshold commonly adopted in microbiome MR studies to retain sufficient instruments given the generally low heritability of microbial traits (Ye et al., 2023; Yu et al., 2025). Instrument strength was assessed using the F-statistic, calculated as F = (N - 2) × *R* ^2^/(1 - *R* ^2^), where N represents the sample size. Only SNPs with F > 10 were retained as instrumental variables to minimize weak instrument bias. In addition, invalid exposure factors were excluded according to the SNPs <2. Subsequently, the harmonise data function in the ‘TwoSampleMR’ package (v 0.6.6) (Freuer and Meisinger, 2023) was utilized to harmonize allele effects and corresponding effect magnitudes.

### 2.3 MR analysis

Various MR approaches, including MR Egger (Xian et al., 2023), weighted median (Liu et al., 2023a), inverse variance weighted (IVW) (Mounier and Kutalik, 2023), weighted mode (Xu et al., 2022), and simple mode (Dai et al., 2023), were utilized to validate the causal correlations between the exposure factors and outcome, and the results mainly relied on the IVW method (*P* < 0.05). Additionally, three types of graphical representations (scatter plots, forest plots, and funnel plots) were created to display the MR findings. Additionally, heterogeneity analysis was performed utilizing the mr_heterogeneity function from the ‘TwoSampleMR’ package (v 0.6.6) (Freuer and Meisinger, 2023). The fixed-effect IVW model was selected for MR analysis since no heterogeneity was detected (*P* > 0.05). Conversely, the random-effects IVW model was chosen for MR analysis since heterogeneity was identified (*P* < 0.05). As demonstrated by the mr_pleiotropy_test function (*P* > 0.05), no significant horizontal pleiotropy was detected. The leave-one-out (LOO) test was also carried out via the mr_leaveoneout function to assess the robustness and reliability of the MR results. To validate that the results obtained from the MR analysis were directionally correct, the Steiger filtering test was performed (*P* < 0.05, Steiger direction = TRUE). After that, genes that passed directionality testing were chosen as key GM. Finally, the SNPs of key GM were mapped to genes through the ‘gprofiler2’ package (v 4.4.2) (Kolberg et al., 2020). Specifically, the gsnpense () function was used, which maps SNPs to Ensembl genes with variant effect annotations based on the Ensembl Variation database without requiring pre-specified genomic distance thresholds (Kolberg et al., 2023). These genes were denoted as gut microbiota-related genes (GM-RGs). This mapping strategy is grounded in the widely accepted premise that most noncoding GWAS signals exert their effects through regulation of nearby gene expression (Mostafavi et al., 2023). Although not all regulatory mechanisms are captured, this approach provides a genetically anchored, unbiased initial candidate gene set for integration with PD transcriptomic data. MR analyses were performed for each taxon individually as an exploratory screening step, without strict multiple testing correction, to avoid discarding potentially relevant taxa for downstream validation.

### 2.4 Differential expression analysis

To discover differentially expressed genes (DEGs) between disease and control samples within the training set, the ‘limma’ package (v 3.54.1) (Ritchie et al., 2015) was employed for differential expression analysis, and DEGs were screened based on P < 0.05 and |log_2_ fold change (FC)| > 0.5. Following this, the ‘ggplot2’ package (v 3.4.1) (Gustavsson et al., 2022) was utilized to construct a volcano plot to display the DEGs, and the ‘ComplexHeatmap’ package (v 2.14.0) (Gu et al., 2016) was used to generate a DEGs heatmap.

### 2.5 Identification and functional elucidation of candidate genes

To identify the DEGs linked to GM in PD, the ‘ggvenn’ package (v 0.1.9) (Chen and Boutros, 2011) was used to intersect DEGs and GM-RGs, and the intersection genes were used as candidate genes. Next, to assess the functional pathways associated with candidate genes in PD, Gene Set Enrichment Analysis (GSEA) of candidate genes was performed. The reference gene set ‘c2. kegg.v7.4. symbols’ was carefully chosen from the MSigDB. Firstly, Spearman correlation analyses were performed between each candidate gene and all the remaining genes separately to obtain the correlation coefficients. Subsequently, genes were arranged in a descending order according to these coefficients. The sorted data were then utilized to conduct GSEA (|normalized enrichment score (NES)| > 1 and P. adjust <0.05) with the implementation of the ‘clusterProfiler’ package (v 4.2.2) (Wu et al., 2021).

### 2.6 Identification and validation of key genes

In light of the candidate genes obtained above, the candidate feature genes were first screened using the LASSO regression analysis with 10-fold cross-validation via the ‘glmnet’ package (v 4.1.4) (Li et al., 2022b), in the training set. Furthermore, the candidate feature genes were also screened using the SVM-RFE of the ‘e1071’package (v 1.7.13) (Long and Yang, 2020). Then the Venn diagram was constructed using the ‘ggvenn’ package (v 0.1.9) (Chen and Boutros, 2011), and the genes obtained by the 2 algorithms were intersected and used as feature genes.

To observe the expression levels of the feature genes within the validation set and training set, the transcriptional levels of the feature genes in the different samples were analyzed through the Wilcoxon test in both datasets (*P* < 0.05). Subsequently, the genes whose expression levels differed across the different samples and whose expression trends were consistent in both datasets were selected and named as the key genes.

### 2.7 RNA extraction and reverse transcription

Gingival tissue samples were collected from healthy controls (H, n = 6) and periodontitis patients (PD, n = 6). [Ethics approval: The study protocol was approved by the Ethics Committee of Chinese PLA General Hospital (approval No. S2025-921-1), and written informed consent was obtained from all participants.] Total RNA was extracted from gingival tissues using the SteadyPure Universal RNA Extraction Kit (Accurate Biotechnology, Hunan, China) according to the manufacturer’s instructions. RNA concentration and purity were measured with NanoDrop ND-2000 spectrophotometer (Thermo Fisher Scientific, MA, United States of America), and RNA integrity was verified by 1% agarose gel electrophoresis. Subsequently, 1 μg of total RNA was reverse-transcribed into complementary DNA (cDNA) using the Evo M-MLV Reverse Transcription Kit (Accurate Biotechnology, Hunan, China).

### 2.8 qRT-PCR

Gingival tissue samples were collected from patients with periodontitis (PD, n = 6) and healthy individuals serving as controls (H, n = 6). Total RNA was extracted and reverse-transcribed into cDNA prior to qRT-PCR analysis. qRT-PCR was performed using the Hieff UNICON® qRT-PCR SYBR Green Master Mix (Yeasen Biotechnology, Shanghai, China) on a CFX96™ Real-Time PCR System (Bio-Rad, Hercules, CA, United States of America). Each 20-μL reaction comprised 10 μL of 2×SYBR Green Master Mix, 0.5 μM of each forward and reverse primer, and 2 μL of diluted cDNA template. All reactions were performed in duplicate to ensure technical reproducibility. Primer sequences for target genes and the internal reference gene β-Actin are listed in Table 1. Relative gene expression levels were normalized to β-Actin and quantified using the 2^−ΔΔCT^ method.

**TABLE 1:** Primer sequences used for quantitative real-time PCR.

Gene	Forward primer (5′→3′)	Reverse primer (5′→3′)
NID2	GAG​CTG​TAC​CAC​TAC​TCC​GAC​T	TGT​TCT​GGT​GGA​TGC​GGT​AGG​A
SH3D19	GAA​GAC​ACT​GGC​AGA​GTT​CAC​C	AGA​ACG​ACA​GCA​TGA​GGA​GCA​C
IL-19	CGT​TCT​ACG​TGG​ACA​GGG​TGT​T	CAG​TGA​CAC​TGC​CTC​TGT​TCC​T
β-actin	CAC​CAT​TGG​CAA​TGA​GCG​GTT​C	AGG​TCT​TTG​CGG​ATG​TCC​ACG​T

### 2.9 Western blotting

Gingival tissue samples from patients with periodontitis (PD, n = 6) and healthy controls (H, n = 6) were homogenized in RIPA lysis buffer supplemented with protease and phosphatase inhibitors. Protein concentrations were determined using a bicinchoninic acid (BCA) protein assay kit. Equal amounts of protein were resolved by sodium dodecyl sulphate–polyacrylamide gel electrophoresis (SDS-PAGE) and subsequently transferred onto polyvinylidene fluoride (PVDF) membranes. Membranes were blocked with 5% non-fat dried milk in Tris-buffered saline containing 0.1% Tween-20 (TBST) for 1 h at room temperature, followed by overnight incubation at 4 °C with primary antibodies against candidate biomarker proteins and β-Tubulin as a loading control (detailed antibody information is provided in Table 2). After three washes with TBST, membranes were incubated with a horseradish peroxidase (HRP)-conjugated goat anti-rabbit secondary antibody (Beyotime Biotechnology, Shanghai, China) for 1 h at room temperature. Protein bands were visualized by enhanced chemiluminescence (ECL) and imaged using a ChemiDoc™ MP Imaging System (Bio-Rad, Hercules, CA, United States of America). Densitometric quantification was performed with ImageJ software (National Institutes of Health, Bethesda, MD, United States of America), and relative protein expression levels were normalized to β-Tubulin.

**TABLE 2:** Antibodies used for Western blot analysis.

Antibody	Target	Supplier	Catalogue No.	Dilution
Anti-NID2	NID2	Abcam, cambridge, United Kingdom	ab232883	1:2000
Anti-SH3D19	SH3D19	Thermo Fisher scientific, MA, United States	PA5-70805	1:2000
Anti-IL-19	IL-19	Abcam, cambridge, United Kingdom	ab198925	1:2000
Anti-β-Tubulin	β-Tubulin (loading control)	Bioworld technology, United States of America	—	1:5000
Anti-rabbit IgG (HRP)	Secondary antibody	Beyotime Biotechnology, Shanghai, China	A0208	1:1000

### 2.10 Expression levels in human tissue and immune infiltration analysis

The BioGPS database (http://biogps.org/#goto=welcome) was used to analyze the expression patterns of key genes in different human tissues.

To understand the immune infiltration of different samples, the CIBERSORT algorithm (v 0.1.0) (Craven et al., 2021) was employed to understand the distribution of 22 distinct immune cells (Liu et al., 2023b) in different samples (with samples exhibiting *P* > 0.05 being excluded from the analysis). Subsequently, the Wilcoxon test was used (*P* < 0.05). Furthermore, the relationships between differential immune cells and key genes were explored using Spearman correlation analysis (|cor| > 0.3 and *P* < 0.05).

### 2.11 Construction of molecular regulatory network, key gene-disease association analysis, and drug prediction

A molecular regulatory network was constructed to elucidate the complexity and diversity of the regulatory mechanisms governing the expression of key genes. The TFs of key genes were predicted using NetworkAnalyst. Moreover, the miRNAs of key genes were forecasted using the miRDB (https://mirdb.org/). Finally, the TF-mRNA and miRNA-mRNA regulatory networks were assembled by ‘Cytoscape’ software (v 3.7.2) (Shannon et al., 2003).

To probe the potential drugs for treating PD and study the potential associations between key genes and drugs, the DGIdb was employed to predict drugs potentially connected with key genes, and the resulting drug-gene network was visualized.

### 2.12 Single-cell data quality control and high-variability gene screening

To ensure the veracity and reliability of the subsequent data analysis, a comprehensive quality control assessment was performed on the sample data from the single-cell dataset GSE171213 using the ‘Seurat’ package (v 5.0.1) (Pereira et al., 2021). The quality control criteria were established as follows: cells with gene (nCount_RNA) counts fewer than 200 or exceeding 15,000 were excluded, cells with gene expression counts (nFeature_RNA) less than 200 or exceeding 4,000 were removed. Cells with a percentage of mitochondrial genes (percent_mt) higher than 25% were also removed.

To obtain genes with relatively large intercellular coefficient of variation, the NormalizeData function of the ‘Seurat’ package (v 5.0.1) (Pereira et al., 2021) was used to integrate and standardize the data, and then the vst approach was used to extract 2,000 highly variable genes (HVGs). The results were visualized using the VariableFeaturePlot function to show the top 10 genes with the most significant variation.

### 2.13 Cell dimension-reduction clustering, annotation, and cell functional enrichment analysis

The data were scaled using the ScaleData function within the ‘Seurat’ package (v 5.0.1) (Pereira et al., 2021), and then the RunPCA function was employed to compute the principal components for clustering based on the first 2000 HVGs. In addition, the JackStrawPlot function was utilized to ascertain the statistically significant principal components. Finally, the outcome of data dimensionality reduction was visualized using the ElbowPlot function.

Unsupervised clustering analysis was carried out through the FindClusters function. Specifically, the resolution was set to 0.25 to ascertain the quantity of cell clusters. Subsequently, the cell clusters were visualized using the UMAP generated via the RunUMAP function.

To annotate the cells in the cluster analysis, the marker genes were obtained from the literature (Song et al., 2023) and the CellMarker database (http://xteam.xbio.top/CellMarker/), and then the cells were annotated. Subsequently, the Dotplot was generated to illustrate the expression levels of these marker genes within each cell type.

To understand the biological functions in which the cells were involved, functional enrichment analysis was performed for each cell in GSE171213 using the analyze_sc_clusters function of the ‘ReactomeGSA’ package (v 1.12.0) (Griss et al., 2020) (*P* < 0.05). Subsequently, the enrichment results corresponding to cells were visualized via the pathways function.

### 2.14 Cell communication analysis

To explore the interactions among cells, an analysis was conducted on ligand-receptor pairs and molecular interactions using the ‘CellChat’ package (v 1.6.1) (Jin et al., 2021) (*P* < 0.05). This analysis presented the intercellular communication networks and receptor-ligand interaction probability point plots.

### 2.15 Identification of key cells and pseudotiming analysis

To identify the key cells between the PD and control groups, in GSE171213, cell types with substantial differences in proportion between the samples identified by chi-square test (*P* < 0.05) were recorded, as well as the critical cell types in PD reported in the existing literature. Pseudotime analysis of key cells was performed using the ‘Monocle2’ package (v2.26.0) (Trapnell et al., 2014). Initially, the dimensionality of the key cells was reduced via the DDRTree algorithm. The clusterCells function was used for clustering, with a resolution of 0.4. Additionally, to focus on the expression of key genes in different cellular developmental states, the plot_gene_in_pseudotime function was used to map the expression trends of key genes in subsets of cells across various developmental states. Finally, the expression heatmap of key genes in subsets of cells in different developmental states was visualized.

### 2.16 Statistical analysis

Bioinformatics analyses were conducted by employing the R programming language (v 4.1.0). Unless otherwise specified, differences between two groups were evaluated using the Wilcoxon test, correlations were assessed by Spearman’s rank correlation test, and cell proportion differences were compared using the chi-square test. Where applicable, multiple-testing correction was performed using the Benjamini–Hochberg false discovery rate (FDR) method, and the resulting adjusted *P* values (*p*.adj) < 0.05 were considered statistically significant; otherwise, a nominal *P* < 0.05 was considered statistically significant. Biological experimental data were analyzed using GraphPad Prism version 10.0 (GraphPad Software, CA, United States of America). All data are presented as mean ± SD. Comparisons between healthy control and periodontitis groups were performed using unpaired Student’s t-test. *P* < 0.05 was considered statistically significant.

## 3 Results

### 3.1 A total of 106 GM-RGs causally related to PD

The F-statistics for all retained instrumental variables ranged from 14.59 to 88.43, all exceeding the conventional threshold of 10, indicating that weak instrument bias was unlikely to affect the causal estimates (Supplementary Table S1). Subsequently, based on the eligible exposure factors, the IVW algorithm was utilized to obtain exposure factors that exhibited a notable causal relationship with PD. A total of 22 exposure factors with a significant causal relationship with PD were obtained, and there were 12 protective factors (odds ratio (OR) < 1), 10 risk factors (OR > 1) (Table 3). The scatter plot demonstrated a negative correlation between 12 exposure factors and PD (slope <0), while other exposure factors and PD were positively correlated (slope >0). Notably, the intercepts were all near 0, indicating that they were not significantly affected by confounding factors (Supplementary Figure S1). The forest plot also indicated that the effect sizes of 12 exposure factors were less than 0, and the effect sizes of 10 exposure factors were greater than 0. Therefore, the 12 exposure factors were protective factors for PD, while 10 exposure factors were risk factors for PD (Supplementary Figure S2). The funnel plot demonstrated that the instrumental variables for the 22 exposure factors were symmetrically distributed, consistent with the absence of directional pleiotropy and supporting the validity of the MR assumptions (Supplementary Figure S3). To rate the dependability of the MR results, a sensitivity analysis was implemented. In the heterogeneity test, the 22 exposur*e*s exhibited a P > 0.05, which indicated the absence of heterogeneity between these exposures and the resultant outcome (Table 4). Moreover, the P corresponding to the horizontal pleiotropy was above 0.05, which indicated the absence of any confounding factor interference (Table 5). The LOO analysis demonstrated that there was no appreciable divergence in the effect size of IVs (Supplementary Figure S4). This finding implied that the outcomes obtained from the MR analysis were dependable and could be considered robust evidence for the investigated associations. Finally, the 22 exposure factors all passe*d* the Steiger test (P < 0.05, Steiger direction = TRUE) (Table 6). Consequently, these 22 GM were identified as key GM; the SNPs of key GM were mapped to genes. In total, 106 GM-RGs (Supplementary Table S2) were obtained for the forthcoming in-depth analysis, given that these represent positional annotations rather than functional effectors, necessitating further prioritization through transcriptomic integration and experimental validation, thereby laying the groundwork for further exploration and research into their roles and implications in the context of PD.

**TABLE 3:** Causal associations between gut microbiota (GM) taxa and periodontitis (PD) identified by inverse variance weighted (IVW) Mendelian randomization analysis.

id.outcome	nsnp	or	or_lci95	or_uci95	Exposure
periodontitis	12	1.284	1.007	1.637	class.Erysipelotrichia.id.2147
periodontitis	12	1.284	1.007	1.637	family.Erysipelotrichaceae.id.2149
periodontitis	6	1.281	1.001	1.640	genus.Veillonella.id.2198
periodontitis	10	0.714	0.569	0.896	genus.*Escherichia*.*Shigella*.id.3504
periodontitis	9	1.479	1.113	1.966	genus.Alistipes.id.968
periodontitis	22	0.752	0.645	0.877	phylum.Actinobacteria.id.400
periodontitis	4	1.311	1.067	1.611	genus.Hungatella.id.11306
periodontitis	23	0.807	0.708	0.921	order.Bifidobacteriales.id.432
periodontitis	14	0.793	0.636	0.989	order.Clostridiales.id.1863
periodontitis	7	1.165	1.019	1.333	order.Bacillales.id.1674
periodontitis	12	1.212	1.035	1.419	genus.unknowngenus.id.2041
periodontitis	9	0.800	0.682	0.938	genus.CandidatusSoleaferrea.id.11350
periodontitis	13	0.830	0.706	0.975	genus.Holdemania.id.2157
periodontitis	16	0.832	0.695	0.995	genus.ErysipelotrichaceaeUCG003.id.11384
periodontitis	9	0.735	0.563	0.960	genus.Collinsella.id.815
periodontitis	12	1.284	1.007	1.637	order.Erysipelotrichales.id.2148
periodontitis	23	0.807	0.708	0.921	family.Bifidobacteriaceae.id.433
periodontitis	26	0.776	0.685	0.879	class.Actinobacteria.id.419
periodontitis	10	0.704	0.566	0.877	genus.Sutterella.id.2896
periodontitis	21	0.835	0.716	0.973	genus.Bifidobacterium.id.436
periodontitis	11	1.216	1.051	1.408	genus.Holdemanella.id.11393
periodontitis	12	1.370	1.069	1.755	genus.Ruminiclostridium9.id.11357

**TABLE 4:** Heterogeneity test results for 22 GM taxa using MR-Egger and IVW methods.

Exposure	Q	Q_pval
class.Erysipelotrichia.id.2147	9.509	0.575
family.Erysipelotrichaceae.id.2149	9.509	0.575
genus.Veillonella.id.2198	1.538	0.909
genus.*Escherichia*.*Shigella*.id.3504	9.248	0.415
genus.Alistipes.id.968	1.381	0.995
phylum.Actinobacteria.id.400	20.061	0.517
genus.Hungatella.id.11306	3.621	0.305
order.Bifidobacteriales.id.432	26.503	0.231
order.Clostridiales.id.1863	9.814	0.709
order.Bacillales.id.1674	3.055	0.802
genus.unknowngenus.id.2041	10.364	0.498
genus.CandidatusSoleaferrea.id.11350	8.000	0.433
genus.Holdemania.id.2157	12.032	0.443
genus.ErysipelotrichaceaeUCG003.id.11384	13.424	0.570
genus.Collinsella.id.815	2.938	0.938
order.Erysipelotrichales.id.2148	9.509	0.575
family.Bifidobacteriaceae.id.433	26.503	0.231
class.Actinobacteria.id.419	26.690	0.372
genus.Sutterella.id.2896	6.496	0.689
genus.Bifidobacterium.id.436	33.556	0.029
genus.Holdemanella.id.11393	9.499	0.486
genus.Ruminiclostridium9.id.11357	12.247	0.345

**TABLE 5:** Horizontal pleiotropy test results for GM-PD associations using MR-Egger intercept analysis.

Exposure	egger_intercept	se	pval
class.Erysipelotrichia.id.2147	−0.019	0.035	0.604
family.Erysipelotrichaceae.id.2149	−0.019	0.035	0.604
genus.Veillonella.id.2198	−0.091	0.154	0.589
genus.*Escherichia*.*Shigella*.id.3504	0.024	0.027	0.400
genus.Alistipes.id.968	0.009	0.039	0.821
phylum.Actinobacteria.id.400	0.034	0.024	0.167
genus.Hungatella.id.11306	−0.009	0.092	0.932
order.Bifidobacteriales.id.432	0.009	0.019	0.638
order.Clostridiales.id.1863	0.029	0.032	0.374
order.Bacillales.id.1674	−0.034	0.045	0.484
genus.unknowngenus.id.2041	−0.013	0.024	0.614
genus.CandidatusSoleaferrea.id.11350	−0.084	0.086	0.365
genus.Holdemania.id.2157	−0.014	0.026	0.590
genus.ErysipelotrichaceaeUCG003.id.11384	−0.013	0.022	0.586
genus.Collinsella.id.815	0.013	0.039	0.758
order.Erysipelotrichales.id.2148	−0.019	0.035	0.604
family.Bifidobacteriaceae.id.433	0.009	0.019	0.638
class.Actinobacteria.id.419	−0.004	0.016	0.786
genus.Sutterella.id.2896	0.003	0.031	0.923
genus.Bifidobacterium.id.436	0.000	0.022	0.986
genus.Holdemanella.id.11393	−0.025	0.023	0.313
genus.Ruminiclostridium9.id.11357	−0.044	0.047	0.366

**TABLE 6:** Steiger directionality test results confirming correct causal directions for all 22 GM taxa.

Exposure	snp_r2. exposure	snp_r2. outcome	Correct causal direction	Steiger pval
class.Erysipelotrichia.id.2147	1.42E-02	1.01E-04	TRUE	2.72E-45
family.Erysipelotrichaceae.id.2149	1.42E-02	1.01E-04	TRUE	2.72E-45
genus.Veillonella.id.2198	9.43E-03	5.28E-05	TRUE	4.73E-31
genus.*Escherichia*.*Shigella*.id.3504	1.58E-02	8.74E-05	TRUE	2.73E-51
genus.Alistipes.id.968	1.13E-02	5.65E-05	TRUE	3.75E-37
phylum.Actinobacteria.id.400	4.00E-02	1.47E-04	TRUE	1.09E-132
genus.Hungatella.id.11306	4.55E-03	1.33E-05	TRUE	2.11E-16
order.Bifidobacteriales.id.432	5.67E-02	1.26E-04	TRUE	7.64E-195
order.Clostridiales.id.1863	1.94E-02	1.12E-04	TRUE	1.76E-62
order.Bacillales.id.1674	9.07E-03	3.96E-05	TRUE	1.84E-30
genus.unknowngenus.id.2041	1.41E-02	6.25E-05	TRUE	1.10E-46
genus.CandidatusSoleaferrea.id.11350	1.57E-02	8.49E-05	TRUE	5.13E-51
genus.Holdemania.id.2157	1.93E-02	1.03E-04	TRUE	3.04E-62
genus.ErysipelotrichaceaeUCG003.id.11384	2.07E-02	1.06E-04	TRUE	4.00E-67
genus.Collinsella.id.815	1.26E-02	5.79E-05	TRUE	1.86E-41
order.Erysipelotrichales.id.2148	1.42E-02	1.01E-04	TRUE	2.72E-45
family.Bifidobacteriaceae.id.433	5.67E-02	1.26E-04	TRUE	7.64E-195
class.Actinobacteria.id.419	5.61E-02	1.20E-04	TRUE	4.88E-193
genus.Sutterella.id.2896	1.15E-02	5.48E-05	TRUE	7.27E-38
genus.Bifidobacterium.id.436	5.23E-02	1.06E-04	TRUE	4.55E-180
genus.Holdemanella.id.11393	1.68E-02	9.30E-05	TRUE	2.52E-54
genus.Ruminiclostridium9.id.11357	2.19E-02	1.09E-04	TRUE	3.72E-71

### 3.2 There were seven candidate genes associated with GM

**FIGURE 1:**
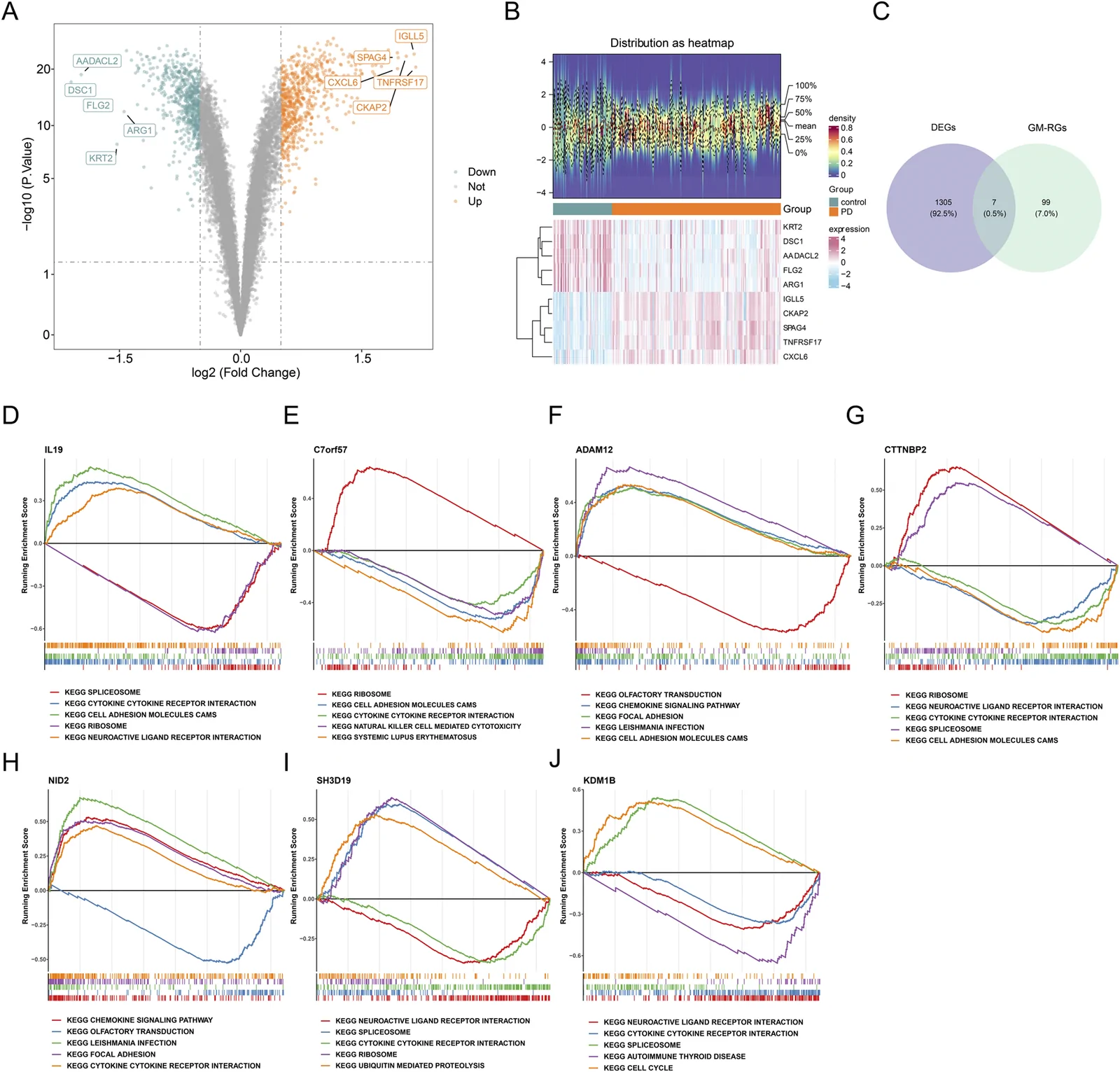
Identification of candidate genes associated with gut microbiota (GM) in periodontitis (PD). **(A)** Volcano plot displaying differentially expressed genes (DEGs) between PD and control samples in the training set (GSE10334). The horizontal axis shows the logarithm of the difference (taking the logarithm of 2), and the vertical axis represents-log10 (P value). Each point in the figure represents a gene, with green points representing significantly downregulated genes, orange points representing significantly upregulated genes, and gray points indicating no significant difference. **(B)** Heatmap of DEGs, illustrating distinct expression patterns in PD versus control samples. In the upper heat map, the color represents the expression density of samples, and the redder the color is, the higher the density is; in the lower heat map, the horizontal coordinate represents samples, and the vertical coordinate represents genes. Red represents high expression genes, and blue represents low expression genes. **(C)** Venn diagram showing the intersection between DEGs and GM-related genes (GM-RGs), yielding seven candidate genes. **(D–J)** Gene Set Enrichment Analysis (GSEA) results for the seven candidate genes, highlighting the top enriched pathways for each. The horizontal axis is the set of genes to be tested in order according to gene correlation, and the vertical axis is the corresponding Running ES. The peak of the line graph is the enrichment score of the enriched pathway.

There were 1,312 DEGs between the patients and controls in the training set, including 657 upregulated genes and 655 downregulated genes (Figures 1A,B). To further identify DEGs associated with GM, 1,312 DEGs and 106 GM-RGs were intersected, and seven intersection genes were obtained as candidate genes (IL-19, C7orf57, ADAM12, CTTNBP2, NID2, SH3D19, and KDM1B) (Figure 1C). GSEA results for the seven candidate genes showed that IL-19 was enriched in 66 pathways. The top 5 pathways were spliceosome, cytokine-cytokine receptor interaction, cell adhesion molecules, ribosome, and neuroactive ligand receptor interaction (Figure 1D; Supplementary Table S3); C7orf57 was enriched in 57 pathways, and the top 5 pathways were cytokine-cytokine receptor interaction, cell adhesion molecules, ribosome, and neuroactive ligand receptor interaction (Figure 1E; Supplementary Table S3); ADAM12 was enriched in 65 pathways, and the top 5 pathways were olfactory transduction, chemokine signaling pathway, focal adhesion, leishmania infection, and cell adhesion molecules (Figure 1F; Supplementary Table S3). In addition, CTTNBP2, NID2, SH3D19, and KDM1B were enriched in 62, 71, 68, and 65 pathways respectively, and the top 5 pathways for each were also presented (Figures 1G–J; Supplementary Table S3). The above results suggested that these key genes may influence the progression of PD by modulating the activation of these pathways.

### 3.3 A total of 3 key genes that impact PD were discovered

**FIGURE 2:**
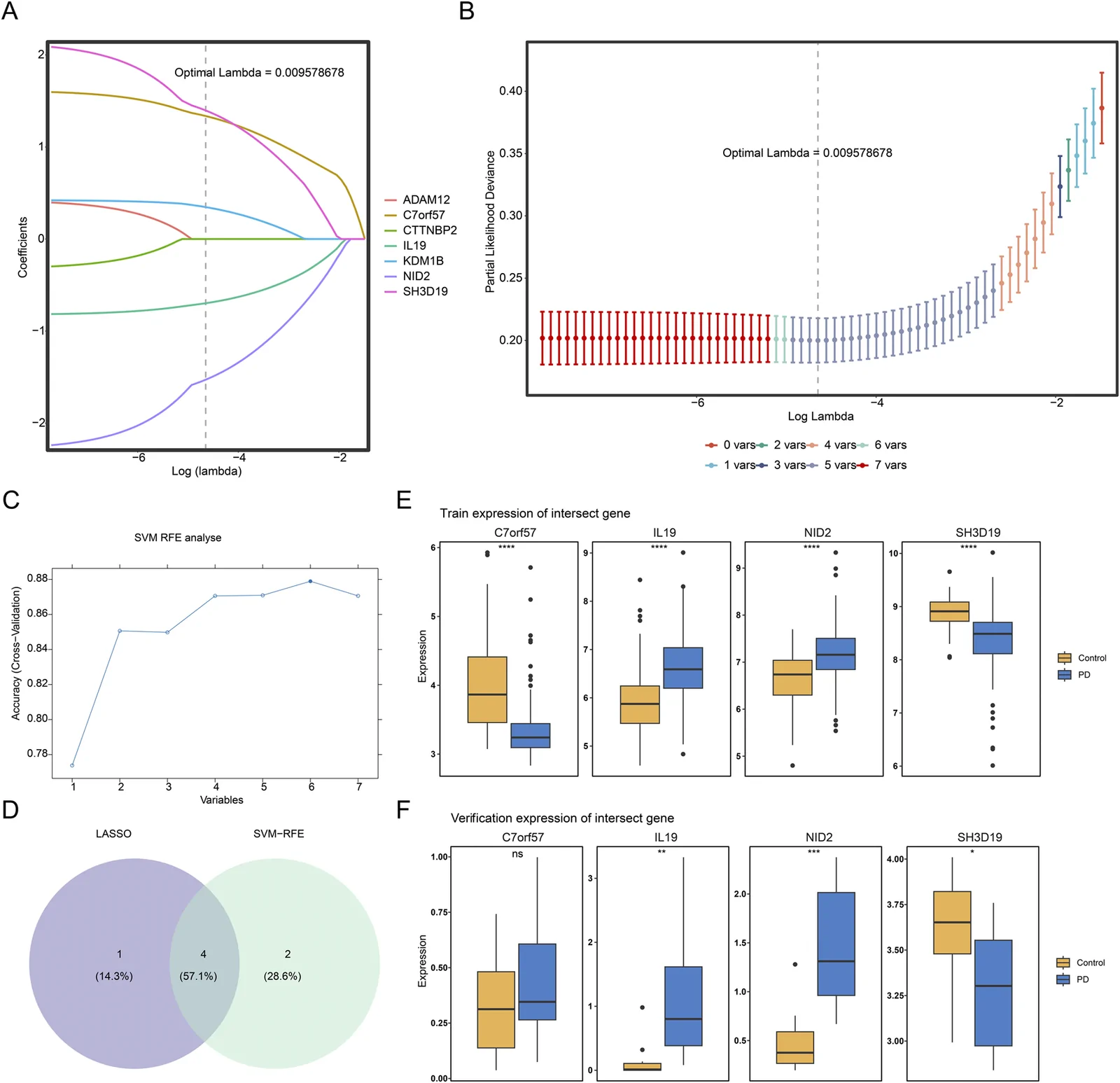
Screening and validation of feature genes in PD. **(A)** LASSO coefficient profiles of candidate genes. The vertical axis is the protein coefficient; the horizontal axis is log (Lambda). **(B)** Cross-validation curve for LASSO regression, indicating the optimal lambda value. The vertical axis represents the cross-validation error, and the red dot represents the mean square error and twice the standard deviation above and below. **(C)** SVM-RFE algorithm performance, showing the ranking of candidate genes. The horizontal axis represents the number of potential characteristic proteins, and the vertical axis represents the generalization error under 10-fold cross validation. **(D)** Venn diagram identifying four overlapping feature genes from LASSO and SVM-RFE analyses. **(E)** Boxplots comparing the expression levels of feature genes between PD and control samples in the training set (GSE10334). **(F)** Validation of feature gene expression in the independent dataset (GSE223924). * indicates that the *P* value is < 0.05; ** indicates that the *P* value is < 0.01; *** indicates that the *P* value is < 0.001; **** indicates that the *P* value is < 0.0001.

Initially, based on the candidate genes, five candidate feature genes were identified using LASSO regression analysis with an optimal lambda value of 0.009,578,678 (Figures 2A,B). In addition, through the SVM-RFE, a total of 6 candidate feature genes were obtained (Figure 2C). Finally, 4 intersection genes of the 2 algorithms (IL-19, C7orf57, NID2, and SH3D19) were obtained, and they were used as feature genes (Figure 2D).

In the training set, these 4 feature genes showed significant differences in expression between PD and control samples (*P* < 0.0001) (Figure 2E). In the validation set, only the IL-19, NID2, and SH3D19 genes showed significant differences between the PD and control samples, with an expression trend consistent with that of the training set (*P* < 0.001) (Figure 2F). Among them, IL-19 and NID2 were upregulated in PD, while SH3D19 showed a downward trend compared with the control. Therefore, IL-19, NID2, and SH3D19 were utilized as key genes.

### 3.4 qRT-PCR and western blot experiments validate significant upregulation of NID2 in periodontitis

**FIGURE 3:**
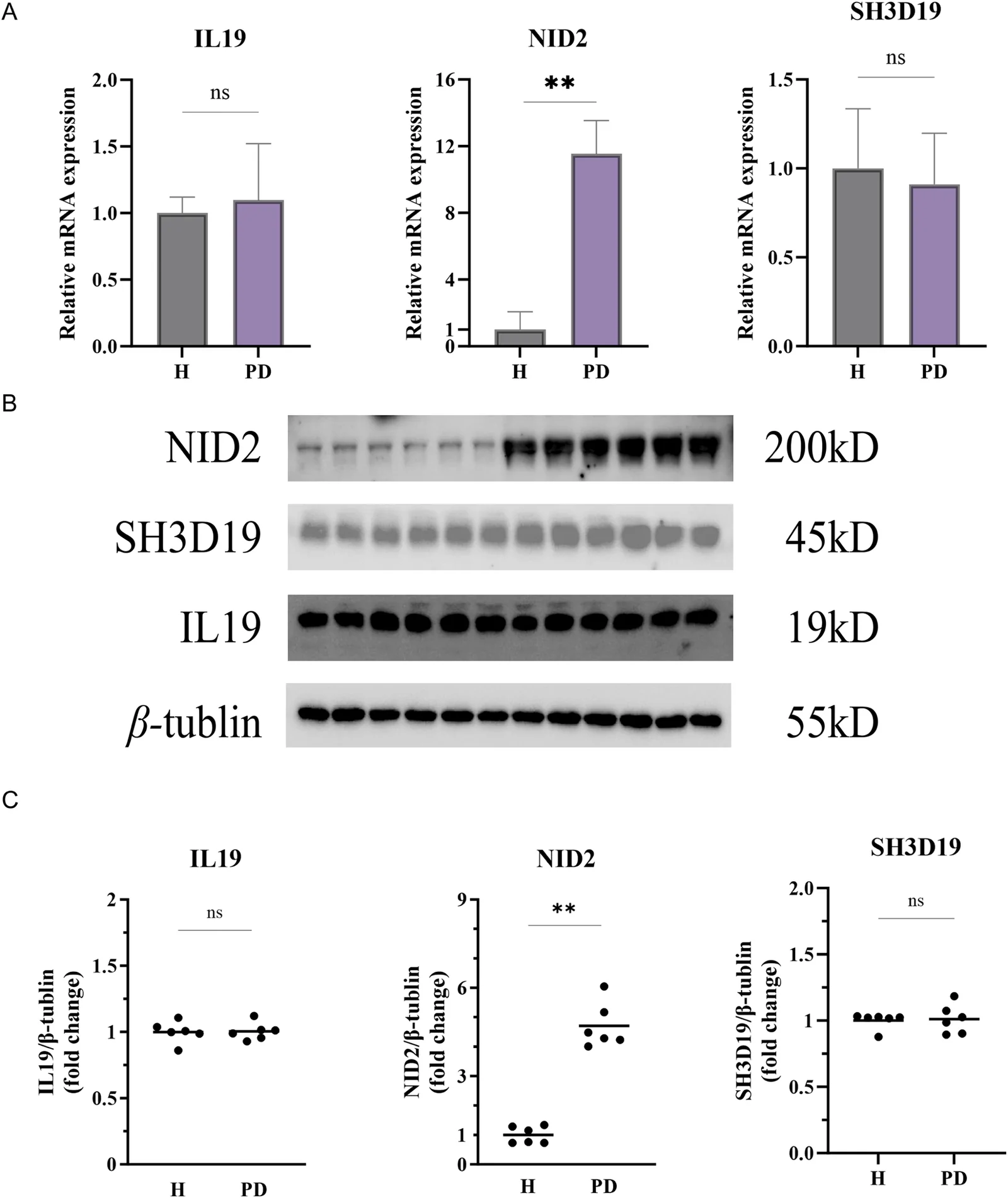
Experimental validation of candidate gene expression in gingival tissue samples from patients with periodontitis and healthy controls. **(A)** Relative mRNA expression levels of NID2, SH3D19, and IL-19 in gingival tissue samples from healthy controls (H, n = 6) and patients with periodontitis (PD, n = 6), as determined by qRT-PCR. Expression levels were normalized to β-actin and calculated using the 2^^−ΔΔCt^ method. Data are presented as mean ± SD. **(B)** Representative Western blot images showing protein expression of NID2 (200 kDa), SH3D19 (45 kDa), IL-19 (19 kDa), and β-tubulin (55 kDa, loading control). **(C)** Quantitative densitometric analysis of Western blot results. Protein expression levels were normalized to β-tubulin and expressed as fold change relative to the healthy control group. Individual data points are shown with horizontal bars indicating the mean. ** indicates that the *P* value is < 0.01; ns, not significant (*P* > 0.05).

Based on the multi-layered screening from previous Mendelian randomization analysis and machine learning algorithms, this study selected three candidate genes (NID2, IL-19, SH3D19) for independent experimental validation. Using gingival tissue samples from the periodontitis group (PD, n = 6) and the healthy control group (H, n = 6), the mRNA expression levels of these genes were detected by qRT-PCR. β-Actin was used as the internal reference gene, and the relative expression of each gene was calculated using the 2^−ΔΔCt^ method. The qRT-PCR results showed that the expression level of NID2 mRNA was significantly higher in the periodontitis group compared to the healthy control group (*P* < 0.01; Figure 3A). IL-19 mRNA showed an upregulating trend in the periodontitis group, but the intergroup difference did not reach statistical significance (*P* > 0.05; Figure 3A). Notably, SH3D19 mRNA exhibited a mild downward trend in the periodontitis group, and the intergroup difference was also not statistically significant (*P* > 0.05; Figure 3A). To further validate the expression changes of the candidate genes at the protein level, Western blot was employed to detect the protein expression of NID2, IL-19, and SH3D19. The results indicated that the expression level of NID2 protein was significantly higher in the periodontitis group compared to the healthy control group (*P* < 0.01; Figure 3B), which was highly consistent with the mRNA-level results. In contrast to NID2, no significant differences were observed in the protein expression levels of IL-19 and SH3D19 between the two groups (Figure 3C). Notably, NID2 was the only gene consistently validated at both the mRNA and protein levels, positioning it as the prioritized candidate for subsequent functional studies. The lack of significant protein-level validation for IL-19 and SH3D19 may be attributable to the limited sample size, post-transcriptional regulatory mechanisms, or the complexity of the local tissue microenvironment. These findings do not exclude their potential involvement but highlight the need for larger cohorts and more refined experimental approaches (e.g., immunohistochemical localization) to clarify their roles. These results should be considered exploratory and warrant independent validation in future large-scale studies.

### 3.5 Key genes influence PD progression through their distribution in different tissues and their correlation with immune cells

**FIGURE 4:**
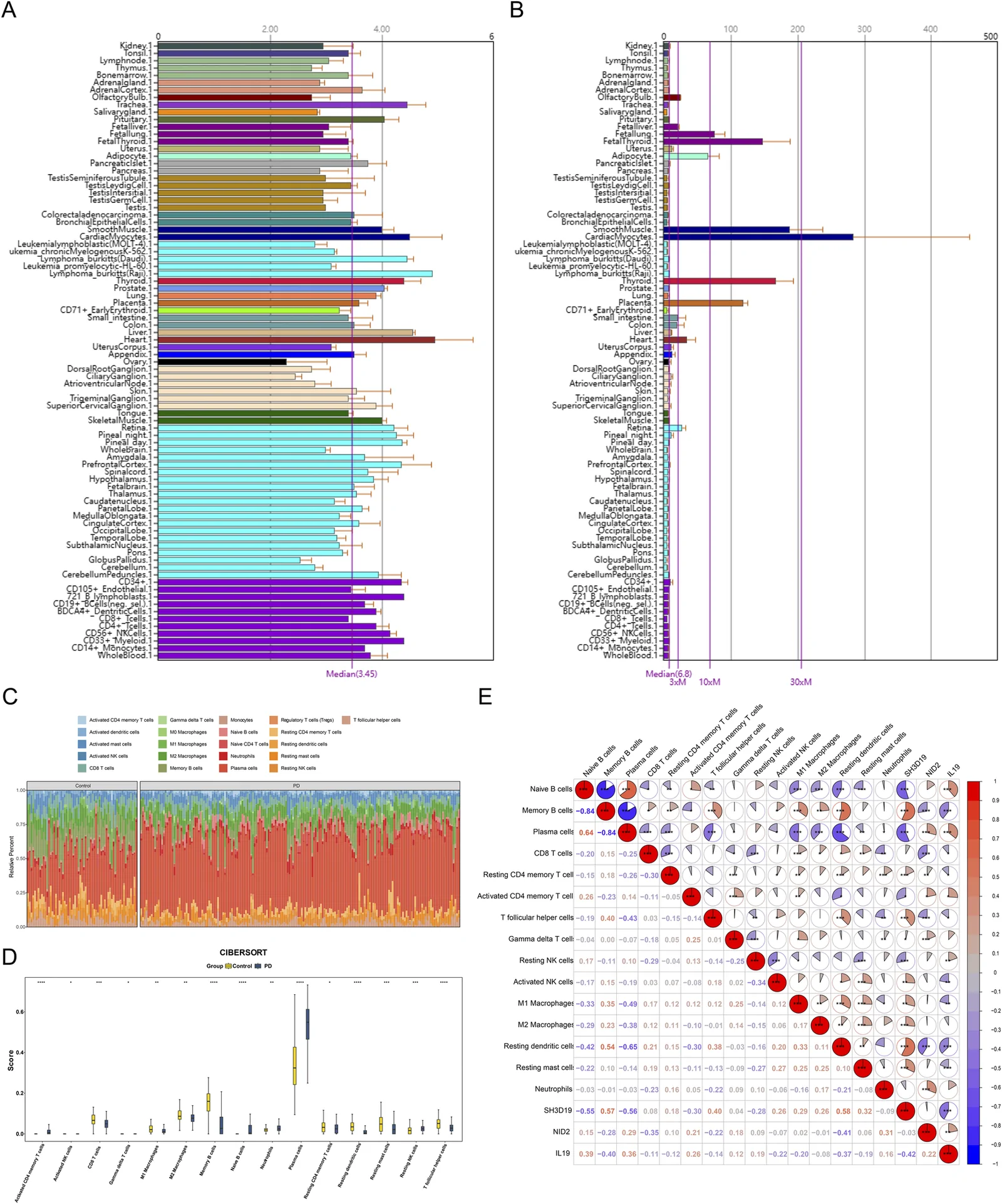
Tissue-specific expression and immune infiltration analysis of key genes. **(A,B)** Bar plots depicting the expression levels of IL-19 and NID2 across human tissues (BioGPS database). Each tissue is represented by the vertical axis and the gene expression in the tissue is represented by the horizontal axis. **(C)** The abundance of different immune cell infiltrations in all samples. **(D)** Boxplots comparing the infiltration levels of significantly altered immune cells. **(E)** Correlation heatmap between key genes and immune cell subsets, with significant associations highlighted.

IL-19 was expressed in various tissues, and the top 3 tissues with the highest expression levels were heart, lymphoma_burkitt’s (Raji), and liver, respectively (Figure 4A). The top 3 tissues with the highest expression levels of NID2 were cardiac myocytes, smooth muscle, and thyroid, respectively (Figure 4B). The expression information of SH3D19 was not recorded in the database.

The proportional distribution of 22 immune cells in PD and control was shown in Figure 4C. There were 15 differentially abundant immune cells between PD and control, including CD8^+^ T cells, neutrophils, plasma cells, and so on. The outcomes revealed that compared with the control, the PD patients exhibited a greater proportion of plasma cells, a lower proportion of CD8^+^ T cells (*P* < 0.001) (Figure 4D). Among them, plasma cells and naive B cells showed the highest positive correlation (cor = 0.64); memory B cells showed the most pronounced negative correlation with both naive B cells and plasma cells (cor = −0.84) (*P* < 0.001) (Figure 4E). Furthermore, SH3D19 had the most notable positive correlation with resting dendritic cells (cor = 0.58), while NID2 had the most pronounced negative correlation with resting dendritic cells (cor = −0.41), IL-19 had the most notable negative correlation with memory B cells (cor = −0.40) (*P* < 0.001) (Figure 4E). The above results indicated that key genes may modulate the immune microenvironment in PD, offering a reference for clinical decision-making in PD management.

### 3.6 Multiple molecules and drugs might be related to the key genes in PD

**FIGURE 5:**
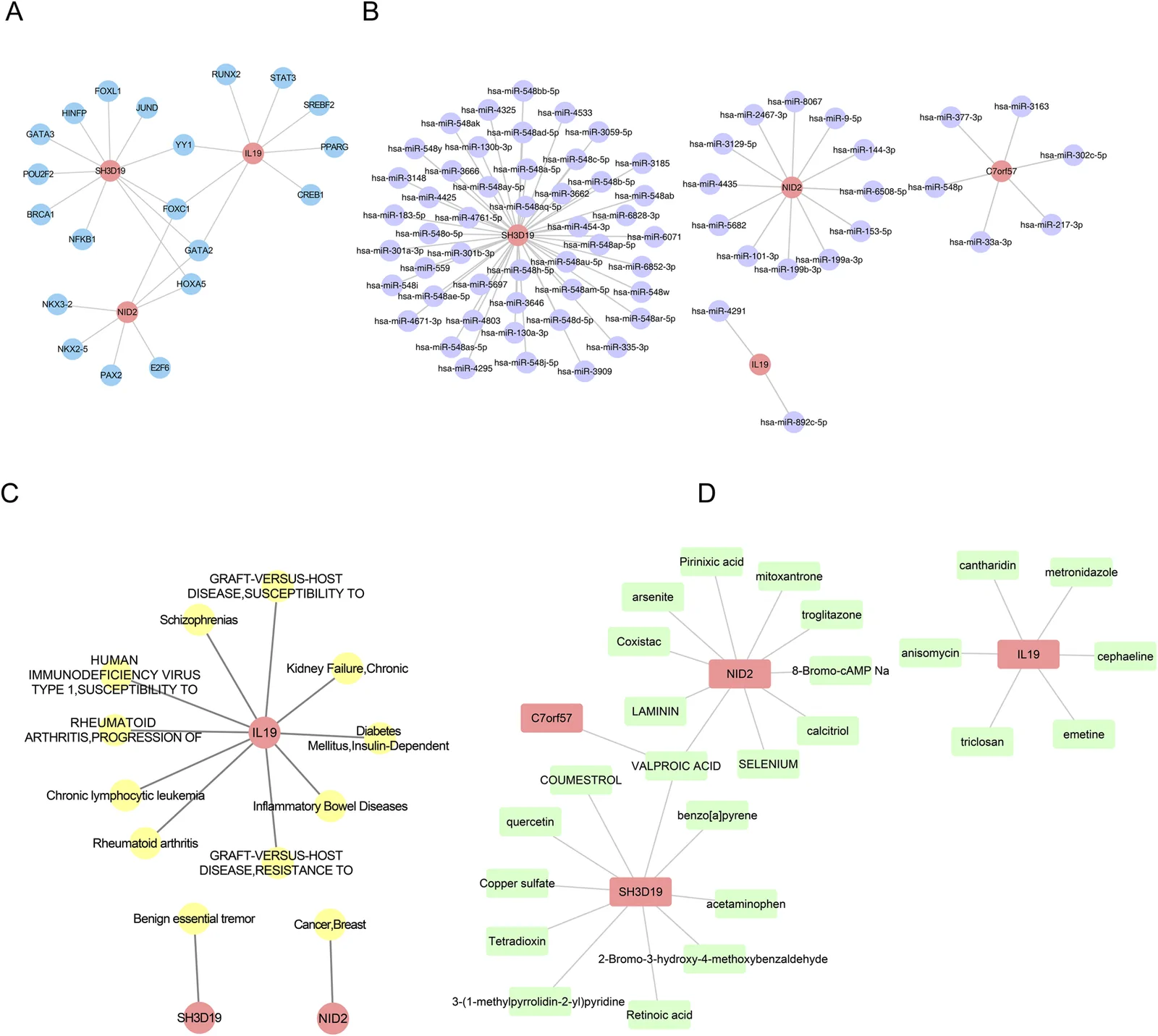
Regulatory networks and drug prediction for key genes. **(A)** TF-mRNA interaction network. Red is the biomarker and blue is the transcription factor. **(B)** miRNA-mRNA network, Red is the biomarker and purple is the miRNA. **(C)** Gene-disease association network, Red is the biomarker and yellow is the disease name. **(D)** Drug-gene interaction network. The square red is the biomarker and the square blue is the transcription factor.

The results of the TF-mRNA regulatory network showed that a total of 20 TFs regulated the expression of key genes. Among them, 3 TFs jointly regulated the expression of both SH3D19 and IL-19, such as YY1, FOXC1, and GATA2 (Figure 5A). A total of 48 miRNAs, such as hsa-miR-4803 and hsa-miR-3662, were predicted to target SH3D19; 12 miRNAs, such as hsa-miR-3129-5p and hsa-miR-9-5p, were predicted to target NID2; and 2 miRNAs, hsa-miR-4291 and hsa-miR-892c-5p, were predicted to target IL-19 (Figure 5B). The gene-disease network revealed that the key genes associated with PD were also linked to various diseases, including immune system disorders (rheumatoid arthritis, chronic lymphocytic leukemia) and psychiatric disorders (schizophrenia), among others (Figure 5C). Based on the DGIdb, the potential drugs of key genes were predicted. In total, 25 drugs were forecast for key genes, such as triclosan, cantharidin, and cephaeline. Among them, valproic acid was the common drug for NID2 and SH3D19 (Figure 5D).

### 3.7 Ten cell types were annotated altogether in GSE171213

**FIGURE 6:**
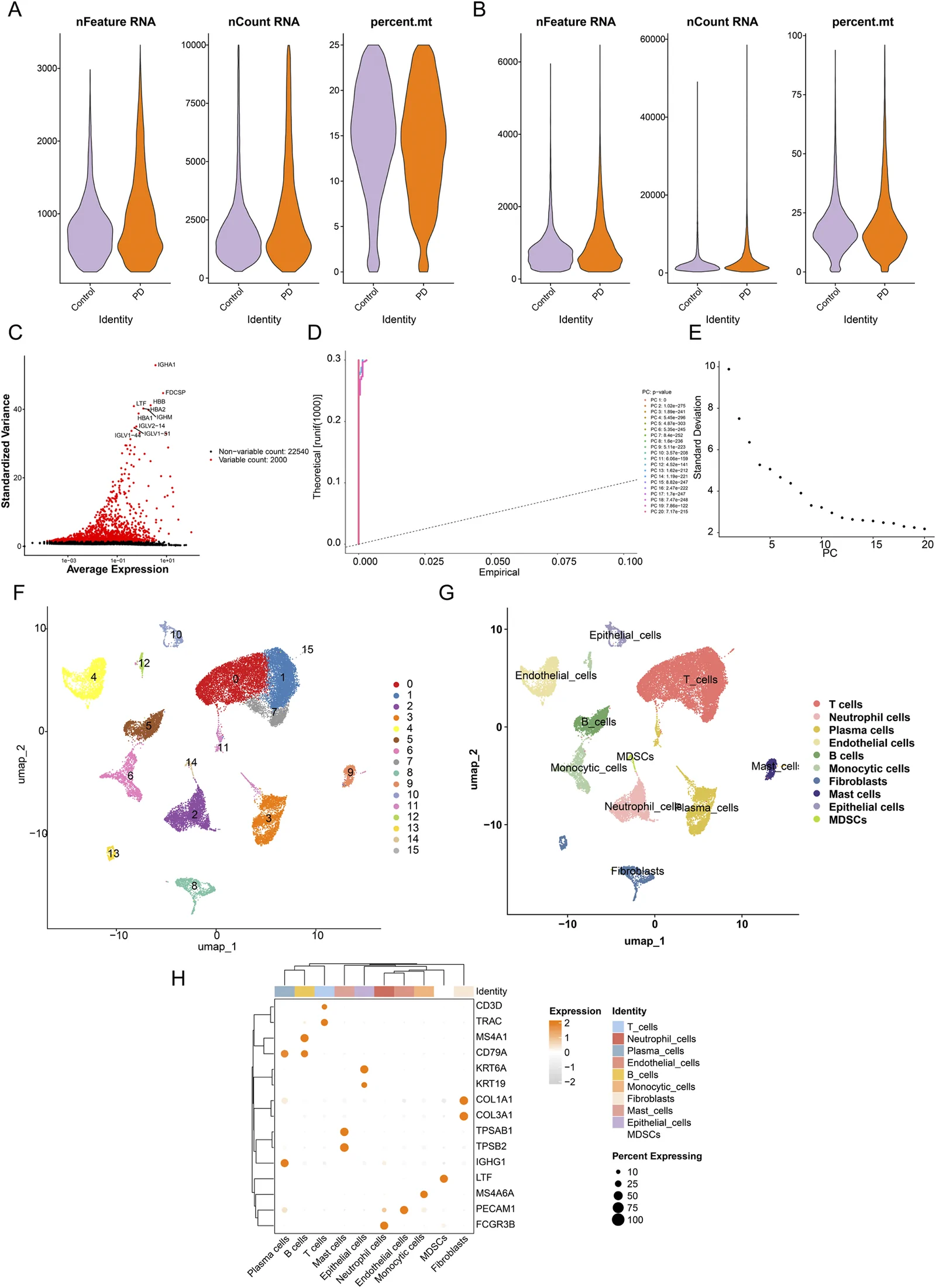
Single-cell RNA sequencing (scRNA-seq) analysis of gingival tissues. **(A,B)** Quality control metrics (nFeature_RNA, nCount_RNA, percent. mt) before” and after filtering. nFeature_RNA represents the number of genes detected in each cell, nCount represents the sum of expression levels of all genes detected in each cell, and percent. mt represents the proportion of mitochondrial genes detected. **(C)** Highly variable genes (HVGs) ranked by variance, with top 10 genes labeled. **(D)** JackStraw plot for significant principal component analysis (PCA) dimensions. **(E)** Elbow plot determining the optimal number of principal components. **(F)** UMAP visualization of 16 unsupervised cell clusters. **(G)** Annotated UMAP plot identifying 10 major cell types. **(H)** Dot plot showing marker gene expression across cell types.

Quality control was performed in the single-cell dataset GSE171213, and after the removal of some cells, the nFeature_RNA, nCount_RNA, and percent. mt distribution maps before and after quality control were displayed (Figure 6A). Thereafter, 2000 HVGs were identified, and the top 10 genes with the highest variance were labeled, such as IGHA1, FDCSP, and HBB (Figure 6B). The JackStraw plot showed extremely significant principal components (*P* < 0.05) (Figure 6C). The top 20 principal components with statistical significance were determined according to the inflection point of the Elbow plot for subsequent single-cell analysis (Figures 6D, E). As shown, cells were divided into 16 clusters (Figure 6F); all clusters were annotated as 10 cell types, such as T cells, B cells, plasma cells, endothelial cells, neutrophils, monocytes, fibroblasts, mast cells, epithelial cells, and myeloid-derived suppressor cells (MDSCs) (Figure 6G). And the dot plot depicting the expressions of marker genes in cells was shown in Figure 6H.

### 3.8 Cells might have affected the occurrence of PD by participating in different functional pathways and interactions among cells

**FIGURE 7:**
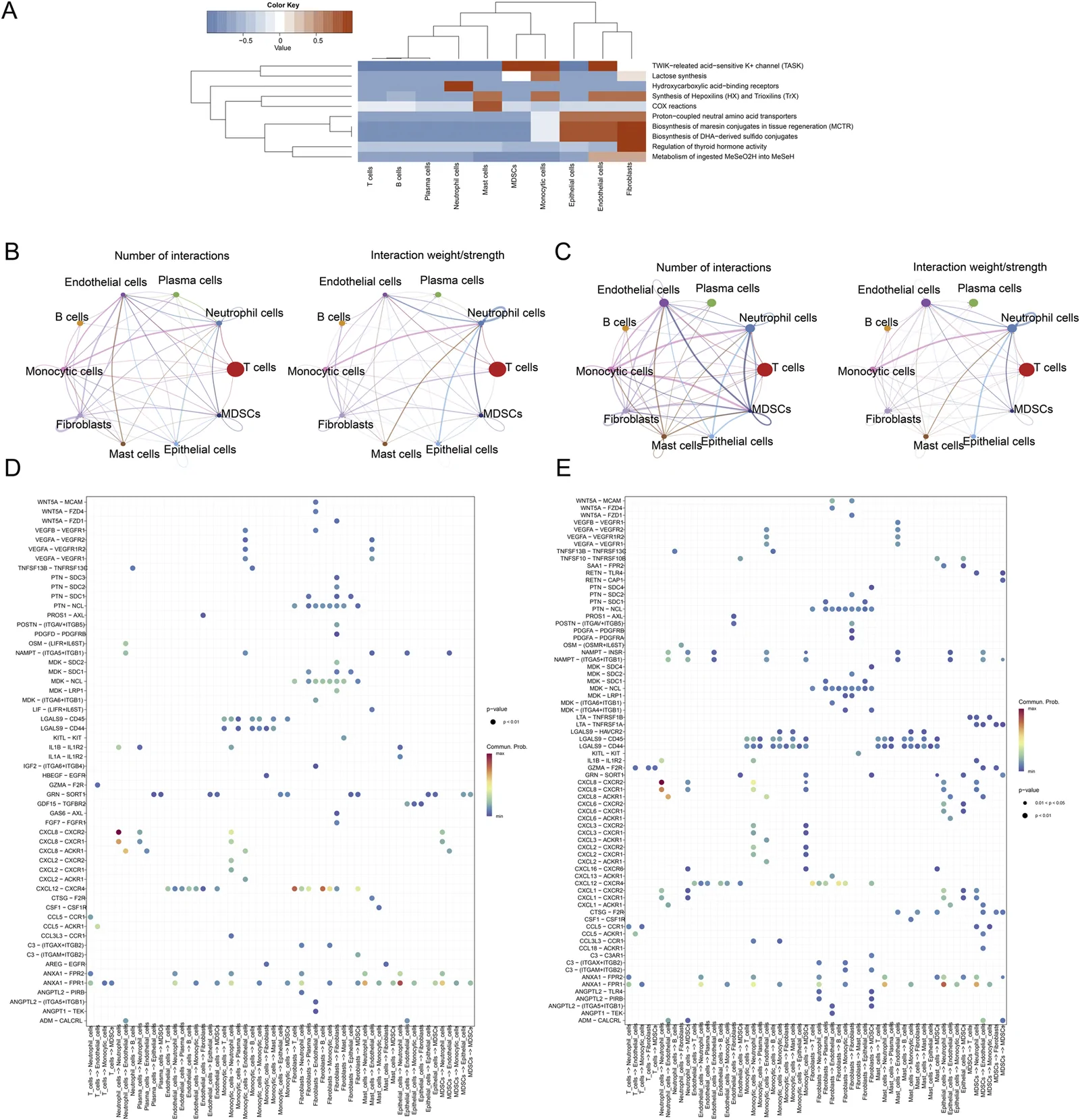
Functional pathways and cell-cell communication in PD. **(A)** Bar plot of the top 10 enriched pathways in annotated cell types (ReactomeGSA). **(B,C)** CellChat analysis of interaction networks: **(B)** Control group, **(C)** PD group, with edge weights indicating interaction strength. **(D,E)** Ligand-receptor interaction probability plots for neutrophils in **(D)** control and **(E)** PD samples. The size of the dots in the figure represents the number of cells, and the thickness of the lines between cells represents the number of interactions between key cells and other cells.

Functional enrichment analysis was performed on annotated cells to understand the biological pathways involved. The top 10 enrichment results showed that endothelial cells were positively correlated with the TWIK-related acid-sensitive K^+^ channels (TASK), synthesis of Hepoxilins (HX) and Trioxilins (TrX), proton-coupled neutral amino acid transporters, biosynthesis of DHA-derived sulfido conjugates, and biosynthesis of maresin conjugates in tissue regeneration (Figure 7A).

The cell communication network diagram showed that in the control group, the number and intensity of interactions between monocytes and neutrophils were the highest (Figure 7B; Supplementary Figure S5A). While in the PD group, apart from the highest number of interactions between monocytes and neutrophils, the numbers of interactions between monocytes and MDSCs, as well as between MDSCs and endothelial cells, and between MDSCs and neutrophilswere also relatively high (Figure 7C; Supplementary Figure S5B). The receptor-ligand interaction diagram showed that in the control samples, when neutrophils served as receptor ligands, the communication probability of CXCL8-CXCR2 was the highest (Figure 7D). In the PD samples, when neutrophils served as receptor ligands, the communication probability of CXCL8-CXCR2 was still the highest (Figure 7E), suggesting that this molecular pair might play an important role in cell-cell interaction.

### 3.9 The key genes might have influenced the disease progression by expressing in different developmental states of the cells

**FIGURE 8:**
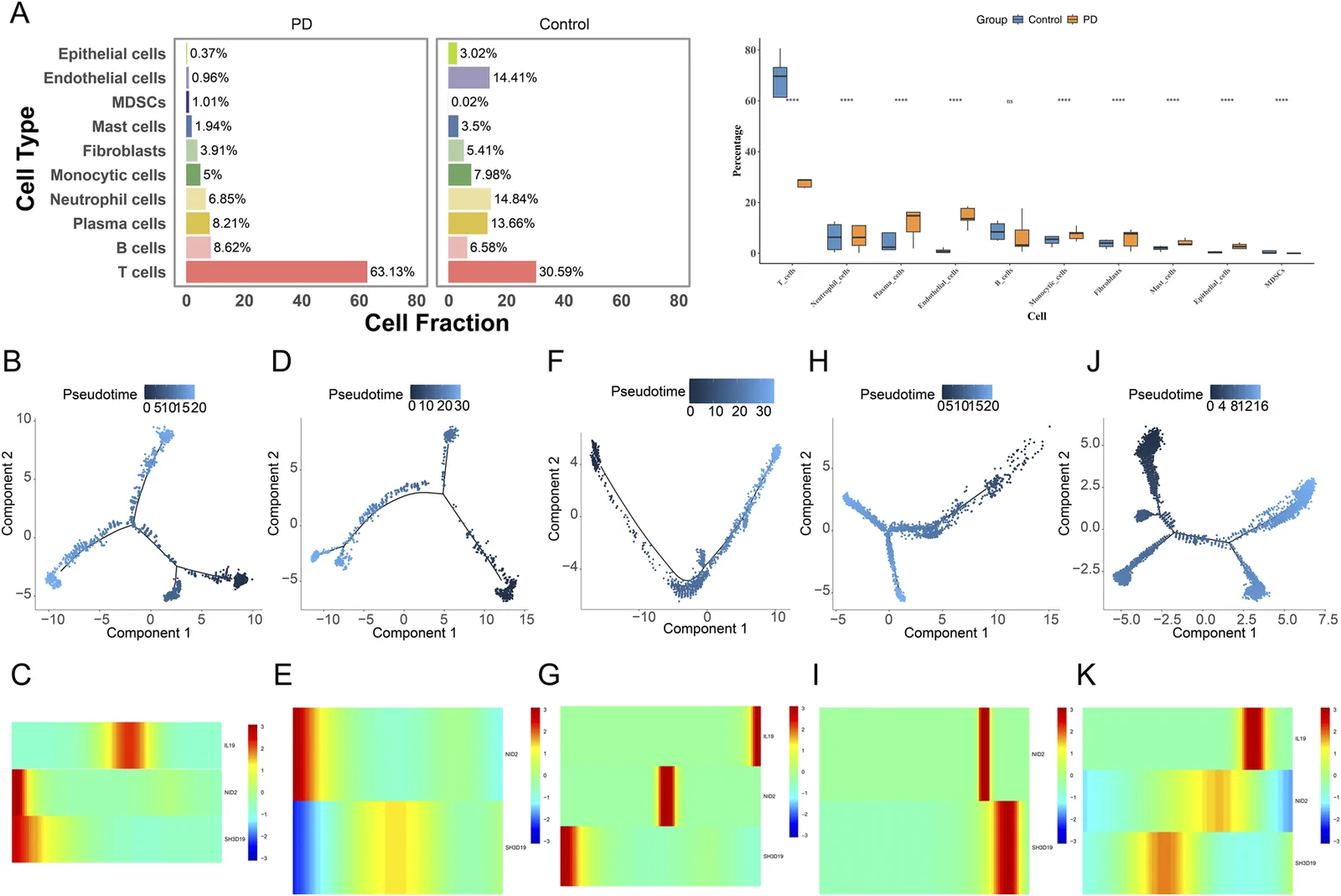
Pseudotime analysis of key cell types in PD. **(A)** Bar plot comparing cell type proportions between PD and control groups and cell proportion difference analysis. **(B,D,F,H,J)** UMAP plots with pseudotime trajectories for endothelial cells, fibroblasts, monocytes, neutrophils, and T cells, colored by developmental states. **(C,E,G,I,K)** Heatmaps displaying dynamic expression patterns of key genes (IL-19, NID2, SH3D19) across pseudotime states for each cell type.

In GSE171213, first of all, the proportions of each annotated cell type in PD and control samples were analyzed. The results revealed that 9 of the 10 annotated cell types showed significant proportional differences between PD and control groups (*P* < 0.05), with the exception of B cells (Figure 8A). The top 3 cell types with the greatest proportion differences were T cells, endothelial cells, and neutrophils. In addition, studies related to PD have revealed the crucial roles of T cells, endothelial cells, neutrophils, fibroblasts, and monocytes. Therefore, a pseudotime analysis was subsequently conducted on these 5 types of cells.

Firstly, after re-dimension reduction, the endothelial cells were clustered into 7 clusters. The cell differentiation process was divided into 5 developmental states. State 1 and state 5 were in the early stage of differentiation, state 2 was in the middle stage of development, and state 3 and state 4 were in the late stage of development. Cluster 1 and cluster 3 were in state 4 and state 3, respectively, and cluster 0 was mostly in the initial stage of development. Notably, the pseudotime trajectory analysis stratified by disease status revealed distinct distribution patterns between PD and control groups. Endothelial cells from control group were predominantly distributed in the early and late stages, whereas cells from PD group were scattered across multiple differentiation stages (Figure 8B; Supplementary Figure S5C). The heatmap of the expression of key genes during the cell development process showed that the genes NID2 and SH3D19 were highly expressed at the beginning of development, and IL-19 was highly expressed in the middle stage of development (Figure 8C).

Fibroblasts were clustered into 7 clusters. Cluster 4 and cluster 3 were mainly in the initial stage of development, while cluster 1 and cluster 2 were mostly in the late stage of development. The cell development process was divided into seven states. State 1 was at the beginning of development, and states 4 and 6 were at the end of development. Stratified analysis showed that fibroblasts in control group were predominantly distributed in the late stage, whereas that in PD group were present in both early and late stages (Figure 8D; Supplementary Figure S5D). During the cell development process, the expression of SH3D19 gradually increased, and NID2 was highly expressed at the beginning of development (Figure 8E).

Monocytes could be clustered into 8 clusters. Cluster 3 and cluster 5 fell within the early stage of differentiation, clusters 4 and 6 were mainly concentrated in the middle stage, cluster 0 was in the late stage, and clusters 1 and 2 were distributed across both middle and late stages. Stratified analysis showed that control monocytes were mainly concentrated in the middle stage, with only a small presence in early and late stages, whereas PD monocytes were predominantly distributed in the middle and late stages (Figure 8F; Supplementary Figure S5E). During the cell development process, IL-19 was highly expressed in the late stage, NID2 was highly expressed in the middle stage, and SH3D19 was highly expressed in the early stage (Figure 8G).

Neutrophils could be clustered into 5 clusters. The entire development process was divided into 5 states. Cluster 3 was mostly in the initial stage of development, corresponding to state 1, and cluster 0 was mostly at the end of development, corresponding to state 4 and state 5. Stratified analysis showed that control neutrophils were mainly concentrated in the middle stage, while PD neutrophils were predominantly distributed in the late stage (Figure 8H; Supplementary Figure S5F). SH3D19 and NID2 were highly expressed near the end of cell development and then decreased (Figure 8I).

T cells could be clustered into 12 clusters. The development process was divided into seven states. Cluster 4 persisted throughout the various stages of cell development. Clusters 5, 6, 7, 8, and 9 were mainly in the early stage of development, while clusters 2 and 0 were in the late stage of development. States 1, 2, and 3 were mainly at the beginning of development, while states 5 and 6 were mostly at the end of development. For T cells, the pseudotime distributions of PD and control groups showed no notable differences (Figure 8J; Supplementary Figure S5G). IL-19 was highly expressed in the middle and late stages of cell development, NID2 was highly expressed in the middle stage of development, and SH3D19 was highly expressed in the early and middle stages of development (Figure 8K).

The above analyses clarified the clustering of each cell type during the development process, the division of states, and the expression patterns of the key genes NID2, SH3D19, and IL-19. These findings provided detailed evidence for a deeper understanding of the dynamic evolution process at the cellular level in PD pathogenesis, offering potential clues for future investigation of disease mechanisms and therapeutic strategies.

## 4 Discussion

Periodontal disease, a type of chronic infectious disease of the periodontal supporting tissues caused by the direct action of pathogenic bacteria, has been confirmed to have a huge impact on both local and systemic health. While GM has been shown to have a connection with PD (Luo et al., 2023), the underlying mechanisms remain poorly understood. While recent MR studies have confirmed causal associations between specific GM taxa and PD (Ye et al., 2023; Yu et al., 2025), they did not explore downstream molecular mediators. By integrating MR with multi-omics and experimental validation, our study identified 22 GM taxa causally associated with PD (10 risk-associated, e.g., Erysipelotrichia, Veillonella, Alistipes; 12 protective, e.g., Actinobacteria, Bifidobacterium, Clostridiales) and prioritized three genes (IL-19, NID2, SH3D19) as potential effector molecules, with NID2 validated at both mRNA and protein levels—providing molecular-level evidence for the oral-gut axis that extends beyond taxonomic-level causality.

IL-19, a member of the IL-10 family, exhibits dual pro-inflammatory and anti-inflammatory properties under different conditions (Ouyang et al., 2011; Small et al., 2021), and can also promote bone resorption by inhibiting osteoprotegerin expression (Chen et al., 2024). This study found that IL-19 expression is significantly upregulated in patients with periodontal disease, which is highly consistent with its pro-bone-resorption function. MR analysis identified the genus Veillonella and the class Erysipelotrichia as risk factors for periodontal disease. The former, as a lactic acid-utilizing bacterium, can lead to intestinal pH and metabolic profile imbalance when its abundance increases (Zhang and Huang, 2023); the latter is associated with metabolic syndrome and chronic low-grade inflammation (Chassaing and Gewirtz, 2014). These risk microbiota can cause metabolic dysregulation (e.g., lactic acid accumulation, increased pro-inflammatory lipopolysaccharides) and activate inflammation by increasing intestinal barrier permeability, thereby upregulating IL-19 expression (Dmytriv et al., 2024; Vardar-Sengul et al., 2009). GSEA enrichment revealed that IL-19 primarily participates in the cytokine-cytokine receptor signaling pathway, suggesting its potential role as an inflammatory signal amplifier. Immunological and pseudo-time series analyses demonstrated that IL-19 interferes with adaptive immune formation and promotes chronic inflammation (Tibbs et al., 2019). This dual effect of sustained inflammation and enhanced bone resorption may suggest a role for IL-19 in linking gut dysbiosis to periodontal inflammation. However, this mechanistic interpretation is hypothesis-generating and requires direct experimental testing (e.g., IL-19 neutralization or overexpression in periodontitis models).

NID2, which maintains the integrity of the basilar membrane, is significantly upregulated in periodontitis and is closely associated with tissue fibrosis and extracellular matrix remodeling (Tracy et al., 2016). MR analysis revealed that Clostridiales, which serve as periodontal protective factors, are reduced in periodontitis. It is known that this bacterial community is a major producer of butyrate in the gut, and its reduction may lead to butyrate deficiency, which could affect histone deacetylase activity (Zhang and Huang, 2023). The removal of epigenetic repression on NID2, a pro-fibrotic gene (Zhao et al., 2025), along with the pro-inflammatory metabolites produced by risk microbiota, further activates fibroblast-to-myofibroblast transformation (Bernardi et al., 2024). Pseudo-time series analysis corroborated this hypothesis: NID2 is highly expressed in early stages of endothelial cells, fibroblasts, and monocytes but subsequently declines, while this downregulation trend is reversed in periodontitis, indicating that dysbiosis-induced loss of epigenetic regulatory factors locks cells in an early pro-fibrotic activation state (Fang et al., 2025). Additionally, NID2 shows a negative correlation with resting dendritic cells (cor = −0.41), suggesting a potential link between NID2 and immune cell function. However, the proposed model—that gut dysbiosis-induced loss of epigenetic regulation locks cells in a pro-fibrotic state—is mechanistically speculative and warrants further validation through functional experiments (e.g., epigenetic assays and loss- or gain-of-function studies).

This study is the first to reveal that SH3D19 is downregulated in periodontal disease, in sharp contrast to the upregulation of IL-19 and NID2, suggesting that it may play a unique role. Immune infiltration revealed a strong positive correlation between SH3D19 and resting dendritic cells (cor = 0.58), while pseudotemporal analysis showed its high expression during the early and middle stages of T-cell, fibroblast, and monocyte development. Given the critical role of the SH3 domain in intracellular trafficking, vesicular transport, and signal transduction (Shimomura et al., 2003; Mehrabipour et al., 2023), the downregulation of SH3D19 may impair the antigen processing and presentation capacity of dendritic cells, weaken local immune surveillance and facilitate pathogen colonization (Marongiu et al., 2022). Transcription factor prediction indicated that YY1, FOXC1, and GATA2 collectively regulate SH3D19 and IL-19. We speculate that the gut microbiota may exert a synergistic regulatory network upstream, downregulating the protective SH3D19 while upregulating the pro-inflammatory IL-19. However, this dual dysregulatory model is hypothesis-generating and requires experimental validation (e.g., TF knockdown/overexpression and functional assays of dendritic cell antigen presentation in the context of SH3D19 downregulation).

Comparing with prior MR findings, our results align with the reported risk effect of Pasteurellales (Ye et al., 2023; Yu et al., 2025). We also identified novel taxa (Veillonella, Erysipelotrichia, Actinobacteria, Bifidobacterium) and extended analyses by mapping SNPs to genes (NID2, IL-19, SH3D19), providing gene-level resolution absent from previous MR-only studies. We acknowledge the potential limitation of tissue heterogeneity, as GM-related genes were derived from SNP mapping (primarily based on blood or fecal samples), while PD-related genes originated from gingival tissues. However, this cross-tissue integration is central to our study design for investigating the oral-gut axis. The oral-gut axis serves as a crucial pathway connecting the oral cavity to systemic health, primarily via ectopic colonization of salivary microbiota, intestinal dysbiosis, and barrier disruption (Zhang et al., 2026). Gut pathogens or metabolites can translocate to distant alveolar bone via circulation and regulate bone homeostasis; gut pathogens can also induce aberrant gut immune responses and subsequent homing of immunocytes to distant organs, contributing to pathological bone loss (Jia et al., 2021). Furthermore, oral dysbiosis can spread to the gut and trigger systemic inflammation, subsequently leading to neuroinflammation and systemic inflammatory conditions (Pravin et al., 2026). Thus, detecting expression changes of GM-RGs in gingival tissues provides key molecular evidence supporting the hypothesis that gut dysbiosis influences periodontal health through systemic pathways.

To further corroborate the reliability of the bioinformatics findings, independent experimental validation of three candidate genes was performed at the clinical sample level. Both qRT-PCR and Western blot analyses revealed that NID2 was significantly upregulated in the periodontitis group compared with healthy controls at both transcriptional and protein levels (*P* < 0.01), which is highly consistent with the bioinformatics results from both the training and validation cohorts. This concordance suggests that the upregulation of NID2 is unlikely to represent a dataset-specific statistical artifact, but rather constitutes a biologically meaningful molecular alteration. The concomitant upregulation at both transcript and protein levels further indicates that the aberrant expression of NID2 is predominantly governed at the transcriptional level, rather than being driven primarily by post-transcriptional modifications, thereby reinforcing its credibility as a candidate biomarker for periodontitis (Chai et al., 2016). By contrast, neither IL-19 nor SH3D19 exhibited statistically significant differences between the two groups at either the mRNA or protein level (*P* > 0.05), which is somewhat discordant with the bioinformatics analyses wherein both genes demonstrated significant differential expression in the validation cohort. This discrepancy might be attributable to several factors. First, the relatively small sample size in the present study (n = 6) affords limited statistical power, rendering it difficult to detect modest effect sizes (Button et al., 2013). The most important limitation of this study is the lack of functional experiments. Although we identified NID2 as a prioritized candidate through multi-omics screening and validated its differential expression in an independent cohort, its precise role in periodontitis pathogenesis—and how it is directly regulated by gut microbial metabolites—remains to be determined through loss- and gain-of-function assays (e.g., gene silencing or overexpression in cell models) and *in vivo* animal studies. Future mechanistic investigations are warranted to establish a causal link between gut dysbiosis, NID2 dysregulation, and periodontal tissue destruction. Second, as a secreted cytokine, IL-19 is subject to complex post-transcriptional regulatory mechanisms within the local microenvironment, potentially resulting in a decoupling between mRNA abundance and protein level (Autieri, 2018). Moreover, the downregulation of SH3D19 in periodontitis was relatively modest in magnitude, which might fall below the threshold of statistical significance under the constraints of a small sample. These findings collectively indicate that the expression dynamics of IL-19 and SH3D19 in periodontitis warrant further validation in larger cohorts, and the present experimental results should not be interpreted as negating their screening value identified through bioinformatics approaches.

We also performed a preliminary *in silico* drug prediction based on the DGIdb database. Among the 25 predicted compounds, valproic acid (VPA) was identified as a common candidate for NID2 and SH3D19. VPA is a classical antiepileptic drug known to inhibit histone deacetylases (HDACs) (Koch-Weser and Browne, 1980; Mishra et al., 2021). Previous studies have reported its potential in periodontal tissue repair, including inhibition of periodontal pathogens and reduction of pro-inflammatory cytokines in rat models (Dong et al., 2022), as well as enhanced osteocalcin and type I collagen expression in newly formed tissues following VPA-preconditioned periodontal ligament stem cell transplantation (Um et al., 2017). However, these reported effects were derived from contexts unrelated to gut microbiota or the specific genes identified in our study. The predicted targeting of NID2 and SH3D19 by VPA is highly speculative and based solely on bioinformatics predictions; it requires rigorous functional validation (e.g., *in vitro* and *in vivo* experiments) before any biological or clinical relevance can be established. We therefore emphasize that these drug predictions are exploratory and should not be overinterpreted.

ScRNA-seq revealed dynamic expression patterns of these genes in specific cell subpopulations (e.g., endothelial cells, fibroblasts, and monocytes). Receptor-ligand analysis revealed that the CXCL8-CXCR2 pathway exhibited the highest communication probability in both groups, indicating its role as the core signaling axis. In periodontitis, monocytes recruit and activate neutrophils through the CXCL8-CXCR2 axis. The latter releases NETs and ROS, which not only eliminate pathogens but also retroactively activate monocytes to secrete IL-1β and TNF-α, forming an inflammatory amplification loop (Jiang et al., 2021). It was found that NID2 and SH3D19 are highly expressed in the late stage of neutrophil development, potentially regulating their effector functions and survival to maintain the inflammatory state. Moreover, cell communication networks indicated that enhanced interactions between monocytes and neutrophils or myeloid-derived suppressor cells (MDSCs) may be a critical feature of PD immunopathology. MDSCs are a heterogeneous population of immature myeloid cells with immunosuppressive functions, which undergo significant expansion in various pathological conditions such as cancer immunity, chronic infections, and autoimmune diseases. MDSCs suppress immune responses through multiple mechanisms, including cytokine secretion, cell surface molecule-mediated intercellular communication, and direct cell-to-cell contact (Hegde et al., 2021). Additionally, MDSCs possess the potential to differentiate into osteoclasts (Li et al., 2024). Relevant studies have demonstrated that the levels of MDSCs in the gingival tissues of periodontitis patients are significantly increased and closely related to the severity of periodontitis (Garcia-Arevalo et al., 2024). On one hand, MDSCs may help suppress excessive immune responses, thereby alleviating periodontal inflammation. On the other hand, their immunosuppressive function might impair host pathogen clearance efficiency. Furthermore, their differentiation into osteoclasts could potentially exacerbate alveolar bone resorption, worsening periodontal destruction. Further research is needed on the subpopulations and phenotypes of MDSCs in periodontitis.

The oral cavity serves as an endogenous reservoir for gut microbiota, where certain opportunistic pathogens persistently disseminate via the oral-gut axis (Schmidt et al., 2019). Dysregulation of the immune response under the influence of these pathogens is a crucial factor in the development of PD (Cekici et al., 2014). By integrating MiBioGen gut microbial genetic data with GWAS and transcriptomic data of PD, this study identified key genes such as IL-19, NID2, and SH3D19 through MR analysis and machine learning. We also identified 15 immune cell types associated with these genes and elucidated the potential pathways of action. This study is the first multi-omics integration study to reveal the causal mechanism by which GM modulates PD progression via IL-19, NID2, and SH3D19, offering a framework for future investigation of potential therapeutic targets.

This study has certain limitations. The MR analysis relies on GWAS data from European populations, and the generalizability of the conclusions to other ethnic groups needs to be verified. The sample size of single-cell data is relatively small (5 PD cases vs. 4 controls), which may affect the statistical power for rare cell subpopulations. Functional mechanisms of the key genes require further validation through *in vitro*/*in vivo* experiments. Future research needs to expand sample diversity, employ organoid or animal models to clarify gene functions, and explore spatiotemporal dynamics of GM-immune microenvironment interactions. MR results were not corrected for multiple comparisons, which may increase false-positive risk; however, downstream transcriptomic integration and experimental validation partially mitigate this limitation. Future studies with larger multi-center cohorts are warranted to confirm the expression differences of these candidate genes. Due to heterogeneity across different study platforms (e.g., distinct internal reference genes, normalization methods, and detection platforms), we were unable to perform a robust meta-analysis based on current publicly available datasets.

## 5 Conclusion

By integrating genomic analysis and multi-omics data, this study investigates the causal relationship between gut microbiota and periodontitis and its underlying molecular mechanisms. Among the identified genes, NID2 emerged as the most promising candidate, independently validated at both mRNA and protein levels. However, whether it functions as a molecular bridge connecting gut dysbiosis to periodontitis pathogenesis requires further functional validation. These findings provide a foundation for future biomarker and therapeutic target development.

## Data Availability

The datasets presented in this study can be found in online repositories. The names of the repository/repositories and accession number(s) can be found in the article/Supplementary Material.
