# Supplementary material for: Investigating the causal impact of gut microbiota on periodontitis based on Mendelian randomization, bulk transcriptomics, and single-cell transcriptomics
Source: Front Cell Dev Biol. 2026 Aug 19;14:1884760. doi: 10.3389/fcell.2026.1884760 (PMC13533959; doi:10.3389/fcell.2026.1884760)
Supplement: Supplementary file 1 [file Table2.docx]

| SNP | exposure | p | Gene_names |
| --- | --- | --- | --- |
| rs2290844 | genus.Alistipes.id.968 | 0.451177828185606 | ADAM12 |
| rs8053479 | genus.ErysipelotrichaceaeUCG003.id.11384 | 0.561727113032446 | ADCY9 |
| rs57024273 | genus.Escherichia.Shigella.id.3504 | 0.956111231904183 | AGAP1 |
| rs13073802 | genus.Veillonella.id.2198 | 0.256441384778551 | ALG1L1P |
| rs59068084 | genus.ErysipelotrichaceaeUCG003.id.11384 | 0.383866924756135 | ALPK1 |
| rs2013594 | genus.Veillonella.id.2198 | 0.216309572194652 | ALX4 |
| rs118526 | genus.Escherichia.Shigella.id.3504 | 0.0795564346626309 | ANKRD34B |
| rs10774377 | order.Clostridiales.id.1863 | 0.235655058006364 | ANO2 |
| rs77293403 | genus.Holdemania.id.2157 | 0.335453129505581 | AP3B1 |
| rs4678258 | genus.CandidatusSoleaferrea.id.11350 | 0.923356999284484 | ARMC8 |
| rs143438747 | genus.Sutterella.id.2896 | 0.218598379916248 | ATG4C |
| rs8130320 | genus.Alistipes.id.968 | 0.581051957736985 | BRWD1 |
| rs2686790 | genus.Bifidobacterium.id.436 | 0.0190729206706982 | C7orf57 |
| rs3792064 | order.Clostridiales.id.1863 | 0.456510523439698 | CAB39 |
| rs1926302 | genus.Holdemanella.id.11393 | 0.983746451572993 | CACHD1 |
| rs35228298 | genus.Holdemanella.id.11393 | 0.583579244948983 | CADM2 |
| rs62113381 | genus.Holdemanella.id.11393 | 0.737329189828418 | CAMSAP3 |
| rs6442336 | order.Clostridiales.id.1863 | 0.429182718691616 | CAND2 |
| rs13020688 | family.Bifidobacteriaceae.id.433 | 0.875098165562135 | CAVIN2-AS1 |
| rs13020688 | genus.Bifidobacterium.id.436 | 0.875098165562135 | CAVIN2-AS1 |
| rs13020688 | order.Bifidobacteriales.id.432 | 0.875098165562135 | CAVIN2-AS1 |
| rs6733130 | order.Bifidobacteriales.id.432 | 0.0341355665497777 | CCNT2-AS1 |
| rs6733130 | genus.Bifidobacterium.id.436 | 0.0341355665497777 | CCNT2-AS1 |
| rs6733130 | family.Bifidobacteriaceae.id.433 | 0.0341355665497777 | CCNT2-AS1 |
| rs74037001 | phylum.Actinobacteria.id.400 | 0.932243557447788 | CMTM5 |
| rs9833771 | phylum.Actinobacteria.id.400 | 0.589224917374131 | CMTM8 |
| rs61825792 | genus.CandidatusSoleaferrea.id.11350 | 0.136296636713432 | CNIH3 |
| rs11958296 | genus.Alistipes.id.968 | 0.932360021555228 | COL23A1 |
| rs76767978 | order.Clostridiales.id.1863 | 0.642526348521462 | CPNE4 |
| rs28568391 | genus.ErysipelotrichaceaeUCG003.id.11384 | 0.0403386355354189 | CPXM2 |
| rs7826267 | family.Erysipelotrichaceae.id.2149 | 0.256360746647831 | CSMD1 |
| rs7826267 | order.Erysipelotrichales.id.2148 | 0.256360746647831 | CSMD1 |
| rs7826267 | class.Erysipelotrichia.id.2147 | 0.256360746647831 | CSMD1 |
| rs10233278 | order.Bacillales.id.1674 | 0.937637573623413 | CTTNBP2 |
| rs80083040 | class.Actinobacteria.id.419 | 0.146444225525407 | CYP7B1 |
| rs687670 | order.Bifidobacteriales.id.432 | 0.091609907824395 | DARS1 |
| rs687670 | phylum.Actinobacteria.id.400 | 0.091609907824395 | DARS1 |
| rs687670 | class.Actinobacteria.id.419 | 0.091609907824395 | DARS1 |
| rs687670 | family.Bifidobacteriaceae.id.433 | 0.091609907824395 | DARS1 |
| rs687670 | genus.Bifidobacterium.id.436 | 0.0916099078243951 | DARS1 |
| rs62403464 | genus.ErysipelotrichaceaeUCG003.id.11384 | 0.372436169013719 | DCDC2 |
| rs17586763 | genus.Holdemanella.id.11393 | 0.00310457005063946 | DLEU1 |
| rs16843660 | genus.unknowngenus.id.2041 | 0.256421011187389 | DNAH7 |
| rs73208162 | genus.Escherichia.Shigella.id.3504 | 0.726404558094531 | DSCR4 |
| rs10164067 | genus.ErysipelotrichaceaeUCG003.id.11384 | 0.954677343370511 | DYM |
| rs961091 | class.Actinobacteria.id.419 | 0.133077229076716 | DYNC1I1 |
| rs16843660 | genus.unknowngenus.id.2041 | 0.256421011187389 | E2F3P2 |
| rs607782 | genus.Holdemanella.id.11393 | 0.22886793418239 | ECI2-DT |
| rs967319 | genus.Holdemania.id.2157 | 0.0330709694816403 | FHIT |
| rs12040548 | genus.Ruminiclostridium9.id.11357 | 0.463755062673139 | GCSAML |
| rs10885477 | genus.Holdemania.id.2157 | 0.765908299854124 | HABP2 |
| rs7400877 | genus.CandidatusSoleaferrea.id.11350 | 0.705760870343896 | IFT43 |
| rs6660520 | class.Actinobacteria.id.419 | 0.0814132084847467 | IL19 |
| rs10781552 | order.Erysipelotrichales.id.2148 | 0.562137564199567 | JAKMIP3 |
| rs10781552 | class.Erysipelotrichia.id.2147 | 0.562137564199567 | JAKMIP3 |
| rs10781552 | family.Erysipelotrichaceae.id.2149 | 0.562137564199567 | JAKMIP3 |
| rs73208162 | genus.Escherichia.Shigella.id.3504 | 0.726404558094531 | KCNJ6 |
| rs214586 | genus.ErysipelotrichaceaeUCG003.id.11384 | 0.509904751293302 | KDM1B |
| rs742016 | genus.Veillonella.id.2198 | 0.805251402704959 | KIAA0930 |
| rs6899771 | order.Bifidobacteriales.id.432 | 0.544337464029581 | KLHL32 |
| rs6899771 | family.Bifidobacteriaceae.id.433 | 0.544337464029581 | KLHL32 |
| rs8003149 | order.Erysipelotrichales.id.2148 | 0.931243188959598 | KTN1 |
| rs8003149 | family.Erysipelotrichaceae.id.2149 | 0.931243188959598 | KTN1 |
| rs8003149 | class.Erysipelotrichia.id.2147 | 0.931243188959598 | KTN1 |
| rs12251396 | genus.ErysipelotrichaceaeUCG003.id.11384 | 0.651332579662523 | LARP4B |
| rs1397793 | phylum.Actinobacteria.id.400 | 0.994678371219379 | LUCAT1 |
| rs6133067 | genus.Holdemania.id.2157 | 0.0135371859083616 | MAVS |
| rs182549 | genus.Bifidobacterium.id.436 | 0.246969248712463 | MCM6 |
| rs182549 | class.Actinobacteria.id.419 | 0.246969248712463 | MCM6 |
| rs182549 | order.Bifidobacteriales.id.432 | 0.246969248712463 | MCM6 |
| rs182549 | phylum.Actinobacteria.id.400 | 0.246969248712463 | MCM6 |
| rs182549 | family.Bifidobacteriaceae.id.433 | 0.246969248712463 | MCM6 |
| rs72767435 | class.Actinobacteria.id.419 | 0.472215896853245 | MCTP2 |
| rs2615052 | genus.Ruminiclostridium9.id.11357 | 0.230653718048209 | MFSD1 |
| rs7234058 | class.Erysipelotrichia.id.2147 | 0.935724482795425 | MIR3976HG |
| rs7234058 | family.Erysipelotrichaceae.id.2149 | 0.935724482795425 | MIR3976HG |
| rs7234058 | order.Erysipelotrichales.id.2148 | 0.935724482795425 | MIR3976HG |
| rs9350083 | genus.Sutterella.id.2896 | 0.0302555937815788 | MIR548A1HG |
| rs80149660 | genus.Holdemania.id.2157 | 0.342805466696679 | MKI67 |
| rs4945008 | class.Actinobacteria.id.419 | 0.890810450314944 | NADSYN1 |
| rs2336448 | genus.unknowngenus.id.2041 | 0.907725535314837 | NCKAP5L |
| rs11207728 | order.Bacillales.id.1674 | 0.399077988948703 | NFIA |
| rs2273429 | order.Clostridiales.id.1863 | 0.504252301648819 | NID2 |
| rs149807560 | genus.Collinsella.id.815 | 0.529485555248016 | NLRP5 |
| rs11591622 | genus.Sutterella.id.2896 | 0.831285417308242 | PAX2 |
| rs13173038 | genus.Sutterella.id.2896 | 0.367996826007873 | PDE4D |
| rs62504403 | family.Erysipelotrichaceae.id.2149 | 0.014656444535444 | PLEKHA2 |
| rs62504403 | order.Erysipelotrichales.id.2148 | 0.014656444535444 | PLEKHA2 |
| rs62504403 | class.Erysipelotrichia.id.2147 | 0.014656444535444 | PLEKHA2 |
| rs9500080 | genus.Holdemania.id.2157 | 0.961421414979768 | PREP |
| rs62376424 | genus.Veillonella.id.2198 | 0.873697494115177 | PRR16 |
| rs12513188 | genus.Holdemanella.id.11393 | 0.532147092707333 | PRR27 |
| rs1867876 | genus.Holdemania.id.2157 | 0.490738163341199 | PTPN5 |
| rs10809135 | genus.CandidatusSoleaferrea.id.11350 | 0.389155366209774 | PTPRD |
| rs7851088 | genus.Hungatella.id.11306 | 0.185496197519329 | PTPRD |
| rs75211493 | phylum.Actinobacteria.id.400 | 0.597466769307542 | PTPRS |
| rs62203853 | genus.CandidatusSoleaferrea.id.11350 | 0.791616619508751 | PTPRT |
| rs1446585 | phylum.Actinobacteria.id.400 | 0.1713998280766 | R3HDM1 |
| rs1446585 | class.Actinobacteria.id.419 | 0.1713998280766 | R3HDM1 |
| rs1446585 | genus.Bifidobacterium.id.436 | 0.1713998280766 | R3HDM1 |
| rs1446585 | family.Bifidobacteriaceae.id.433 | 0.1713998280766 | R3HDM1 |
| rs1446585 | order.Bifidobacteriales.id.432 | 0.1713998280766 | R3HDM1 |
| rs6745996 | order.Bifidobacteriales.id.432 | 0.138515915019872 | R3HDM1 |
| rs6745996 | class.Actinobacteria.id.419 | 0.138515915019872 | R3HDM1 |
| rs6745996 | family.Bifidobacteriaceae.id.433 | 0.138515915019872 | R3HDM1 |
| rs6745996 | phylum.Actinobacteria.id.400 | 0.138515915019872 | R3HDM1 |
| rs6745996 | genus.Bifidobacterium.id.436 | 0.138515915019872 | R3HDM1 |
| rs6730157 | class.Actinobacteria.id.419 | 0.117685562586829 | RAB3GAP1 |
| rs6730157 | family.Bifidobacteriaceae.id.433 | 0.117685562586829 | RAB3GAP1 |
| rs6730157 | order.Bifidobacteriales.id.432 | 0.117685562586829 | RAB3GAP1 |
| rs6730157 | genus.Bifidobacterium.id.436 | 0.117685562586829 | RAB3GAP1 |
| rs6730157 | phylum.Actinobacteria.id.400 | 0.117685562586829 | RAB3GAP1 |
| rs9809789 | genus.Ruminiclostridium9.id.11357 | 0.0458686873393679 | RBMS3 |
| rs9809789 | genus.Ruminiclostridium9.id.11357 | 0.0458686873393679 | RBMS3-AS3 |
| rs290772 | order.Clostridiales.id.1863 | 0.0354690860314693 | RPS6P3 |
| rs4636956 | genus.Holdemania.id.2157 | 0.421992863203169 | RSKR |
| rs58891024 | genus.Ruminiclostridium9.id.11357 | 0.0646976447604803 | RSRC1 |
| rs73166358 | genus.Ruminiclostridium9.id.11357 | 0.299156958540914 | RSRC1 |
| rs73172105 | genus.Ruminiclostridium9.id.11357 | 0.182570762063303 | RSRC1 |
| rs2050185 | genus.Sutterella.id.2896 | 0.920364630894258 | SAMD5 |
| rs6514318 | genus.unknowngenus.id.2041 | 0.858475603683958 | SDCBP2-AS1 |
| rs11655079 | order.Bifidobacteriales.id.432 | 0.418530884071407 | SEPTIN9 |
| rs11655079 | family.Bifidobacteriaceae.id.433 | 0.418530884071407 | SEPTIN9 |
| rs11655079 | class.Actinobacteria.id.419 | 0.418530884071407 | SEPTIN9 |
| rs13249325 | genus.Hungatella.id.11306 | 0.0474703408770025 | SGCZ |
| rs74303178 | genus.Ruminiclostridium9.id.11357 | 0.258337065580864 | SGCZ |
| rs6815608 | order.Clostridiales.id.1863 | 0.34522664414391 | SH3D19 |
| rs1287018 | order.Bacillales.id.1674 | 0.791382573627025 | SHLD1 |
| rs7187855 | genus.unknowngenus.id.2041 | 0.0247823120128429 | SLC7A5 |
| rs11745923 | order.Bifidobacteriales.id.432 | 0.390740610563116 | SLC9A3 |
| rs11745923 | family.Bifidobacteriaceae.id.433 | 0.390740610563116 | SLC9A3 |
| rs11745923 | class.Actinobacteria.id.419 | 0.390740610563116 | SLC9A3 |
| rs11745923 | order.Bifidobacteriales.id.432 | 0.390740610563116 | SLC9A3-AS1 |
| rs11745923 | family.Bifidobacteriaceae.id.433 | 0.390740610563116 | SLC9A3-AS1 |
| rs11745923 | class.Actinobacteria.id.419 | 0.390740610563116 | SLC9A3-AS1 |
| rs11941716 | genus.unknowngenus.id.2041 | 0.206676261154112 | SORCS2 |
| rs13105690 | order.Clostridiales.id.1863 | 0.76780324731458 | SORCS2 |
| rs72738886 | order.Clostridiales.id.1863 | 0.176991288134218 | SPEF2 |
| rs12049045 | class.Actinobacteria.id.419 | 0.451257825126644 | SYT6 |
| rs1145877 | genus.Sutterella.id.2896 | 0.30881180581751 | TENT5A |
| rs2300774 | order.Erysipelotrichales.id.2148 | 0.621373639645867 | TFRC |
| rs2300774 | class.Erysipelotrichia.id.2147 | 0.621373639645867 | TFRC |
| rs2300774 | family.Erysipelotrichaceae.id.2149 | 0.621373639645867 | TFRC |
| rs1961273 | order.Bifidobacteriales.id.432 | 0.288938309148485 | THSD7B |
| rs1961273 | family.Bifidobacteriaceae.id.433 | 0.288938309148485 | THSD7B |
| rs1961273 | class.Actinobacteria.id.419 | 0.288938309148485 | THSD7B |
| rs1961273 | genus.Bifidobacterium.id.436 | 0.288938309148485 | THSD7B |
| rs13020688 | family.Bifidobacteriaceae.id.433 | 0.875098165562135 | TMEFF2 |
| rs13020688 | genus.Bifidobacterium.id.436 | 0.875098165562135 | TMEFF2 |
| rs13020688 | order.Bifidobacteriales.id.432 | 0.875098165562135 | TMEFF2 |
| rs638382 | class.Actinobacteria.id.419 | 0.00688182299145252 | TMEM163 |
| rs638382 | family.Bifidobacteriaceae.id.433 | 0.00688182299145252 | TMEM163 |
| rs638382 | genus.Bifidobacterium.id.436 | 0.00688182299145252 | TMEM163 |
| rs638382 | order.Bifidobacteriales.id.432 | 0.00688182299145252 | TMEM163 |
| rs638382 | phylum.Actinobacteria.id.400 | 0.00688182299145252 | TMEM163 |
| rs34471047 | genus.Hungatella.id.11306 | 0.0149303219529961 | TMEM192 |
| rs11994308 | genus.ErysipelotrichaceaeUCG003.id.11384 | 0.461365025715406 | TMEM64 |
| rs73311093 | genus.ErysipelotrichaceaeUCG003.id.11384 | 0.686760362660683 | TMEM64 |
| rs112767262 | genus.Escherichia.Shigella.id.3504 | 0.198436287540148 | TRAP1 |
| rs1496626 | genus.Collinsella.id.815 | 0.362955959905149 | TSHZ3 |
| rs62102630 | genus.Collinsella.id.815 | 0.623039868988305 | TSHZ3-AS1 |
| rs7400877 | genus.CandidatusSoleaferrea.id.11350 | 0.705760870343896 | TTLL5 |
| rs3213889 | genus.Bifidobacterium.id.436 | 0.164274019358605 | UBXN4 |
| rs3213889 | order.Bifidobacteriales.id.432 | 0.164274019358605 | UBXN4 |
| rs3213889 | class.Actinobacteria.id.419 | 0.164274019358605 | UBXN4 |
| rs3213889 | family.Bifidobacteriaceae.id.433 | 0.164274019358605 | UBXN4 |
| rs3213889 | phylum.Actinobacteria.id.400 | 0.164274019358605 | UBXN4 |
| rs6126494 | order.Clostridiales.id.1863 | 0.72503451172201 | ZFP64 |
| rs10209482 | genus.Bifidobacterium.id.436 | 0.252560828629603 | ZRANB3 |
| rs10209482 | class.Actinobacteria.id.419 | 0.252560828629603 | ZRANB3 |
| rs10209482 | family.Bifidobacteriaceae.id.433 | 0.252560828629603 | ZRANB3 |
| rs10209482 | order.Bifidobacteriales.id.432 | 0.252560828629603 | ZRANB3 |
| rs10209482 | phylum.Actinobacteria.id.400 | 0.252560828629603 | ZRANB3 |
| rs13023281 | phylum.Actinobacteria.id.400 | 0.828381606775848 | ZRANB3 |
| rs13023281 | family.Bifidobacteriaceae.id.433 | 0.828381606775848 | ZRANB3 |
| rs13023281 | genus.Bifidobacterium.id.436 | 0.828381606775848 | ZRANB3 |
| rs13023281 | order.Bifidobacteriales.id.432 | 0.828381606775848 | ZRANB3 |
| rs13023281 | class.Actinobacteria.id.419 | 0.828381606775848 | ZRANB3 |
| rs6730157 | class.Actinobacteria.id.419 | 0.117685562586829 | ZRANB3 |
| rs6730157 | family.Bifidobacteriaceae.id.433 | 0.117685562586829 | ZRANB3 |
| rs6730157 | order.Bifidobacteriales.id.432 | 0.117685562586829 | ZRANB3 |
| rs6730157 | genus.Bifidobacterium.id.436 | 0.117685562586829 | ZRANB3 |
| rs6730157 | phylum.Actinobacteria.id.400 | 0.117685562586829 | ZRANB3 |
